# Promotion of BST2 expression by the transcription factor IRF6 affects the progression of endometriosis

**DOI:** 10.3389/fimmu.2023.1115504

**Published:** 2023-04-18

**Authors:** Jixin Li, Yanan He, Yanjun Qu, Chengcheng Ren, Xiaotong Wang, Yan Cheng, Liyuan Sun, Xin Zhang, Guangmei Zhang

**Affiliations:** ^1^ Department of Gynecology, The First Affiliated Hospital of Harbin Medical University, Harbin, Heilongjiang, China; ^2^ Central Laboratory, The First Affiliated Hospital of Harbin Medical University, Harbin, Heilongjiang, China

**Keywords:** endometriosis, transcription factor, immune-related genes, NF‐κB, lymphangiogenesis, feedback loop

## Abstract

**Background:**

Endometriosis (EM) is a benign, multifactorial, immune-mediated inflammatory disease that is characterized by persistent activation of the NF‐κB signaling pathway and some features of malignancies, such as proliferation and lymphangiogenesis. To date, the pathogenesis of EM is still unclear. In this study, we investigated whether BST2 plays a role in the development of EM.

**Methods:**

Bioinformatic analysis was performed with data from public databases to identify potential candidate targets for drug treatment. Experiments were conducted at the cell, tissue, and mouse EM model levels to characterize the aberrant expression patterns, molecular mechanisms, biological behaviors of endometriosis as well as treatment outcomes.

**Results:**

BST2 was significantly upregulated in ectopic endometrial tissues and cells compared with control samples. Functional studies indicated that BST2 promoted proliferation, migration, and lymphangiogenesis and inhibited apoptosis *in vitro* and *in vivo*. The transcription factor (TF) IRF6 induced high BST2 expression by directly binding the BST2 promoter. The underlying mechanism by which BST2 functions in EM was closely related to the canonical NF‐κB signaling pathway. New lymphatic vessels may serve as a channel for the infiltration of immune cells into the endometriotic microenvironment; these immune cells further produce the proinflammatory cytokine IL-1β, which in turn further activates the NF‐κB pathway to promote lymphangiogenesis in endometriosis.

**Conclusion:**

Taken together, our findings provide novel insight into the mechanism by which BST2 participates in a feedback loop with the NF‐κB signaling pathway and reveal a novel biomarker and potential therapeutic target for endometriosis.

## 1 Introduction

Endometriosis is characterized by the presence of endometrial tissue outside the uterus; it is a common chronic, benign, inflammatory gynecological disease that is dependent on estrogen and affects an estimated 5-10% of women during their reproductive years (1). The typical clinical symptoms of endometriosis include chronic pelvic pain, dysmenorrhea, dyspareunia, and infertility (2). In addition, endometriosis is also a risk factor for clear-cell or endometrioid ovarian cancer (3). The symptoms associated with endometriosis result in 0.809 quality-adjusted life years per woman, and the economic burden of women with endometriosis is similar to the costs associated with other chronic diseases (type 2 diabetes, Crohn’s disease, and rheumatoid arthritis) (4). Recently, increasing evidence suggests that endometriosis affects systems beyond the pelvis, and this disease exerts multifactorial effects throughout the body, such as causing metabolism in certain organs or tissues, leading to systemic inflammation and resulting in pain sensitization and mood disorders (5).

Several theories have been proposed to explain the pathogenesis of endometriosis; the most commonly accepted theory is so-called retrograde menstruation, which was proposed by Sampson (6). According to this theory, a few menstrual tissue fragments, cells, and blood move through the fallopian tubes in a retrograde manner and attach to the peritoneal cavity (7). The immune system is thought to play a major role in the survival of these tissues and cells in the pelvic microenvironment by causing immune tolerance, suppressing immunosurveillance, and preventing their phagocytosis by immune cells (8). Based on multiple lines of evidence, women with endometriosis not only exhibit an altered immune status in the endometrium but also exhibit altered peripheral immune responses (7, 9). The aberrant changes that occur in the peritoneal environment also result in the recruitment of a large number of immune cells, inflammatory-associated proteins, and relevant cytokines, and these factors are all found to be related to the pathophysiology of endometriosis (8, 10). Moreover, further theories regarding hematogenous and lymphatic dissemination have been proposed, and some studies have shown that lymph flows from the uterus to the ovaries, suggesting that the lymphatic system may play a crucial role in the development of endometriosis; these findings also provide favorable evidence that supports the idea that endometriosis is a systemic disease (11, 12). Compared with angiogenesis, much less is known about the role of lymphangiogenesis in the development and progression of endometriosis. The process of lymphangiogenesis mainly involves the migration of lymphatic endothelium from existing lymphatic vessels and the formation of new lymphatic vessels (13). Some data have reported that lymphangiogenesis may play critical roles in the pathogenesis of endometriosis, especially in cases of highly recurrent disease after medical treatment and/or surgical removal (14). Masako Honda“s paper indicated the role of RAMP1 signaling in the regulation of lymphangiogenesis and in the development of endometriosis and provided a promising option for the treatment of endometriosis (15). A further study on PubMed demonstrated that VEGFR1 signaling plays a role in growth and lymphangiogenesis in endometrial tissues and that blockade of VEGFR1 may be a useful approach for the treatment of endometriosis (16). However, the mechanisms by which lymphangiogenesis contributes to the establishment and progression of endometriotic lesions have not yet been fully elucidated.

In our previous study, we combined the Immunology Database and Analysis Portal (ImmPort) database and the Gene Expression Omnibus (GEO) database to acquire a series of differentially expressed immune-related genes (17, 18). Among these genes, we are particularly interested in BST2, which is also known as tethered membrane protein, or CD317, which is a 30-36 kDa interferon-induced type II single channel transmembrane protein and has been previously indicated to trigger the classical inflammatory NF-κB signaling pathway; this pathway is inextricably linked to endometriosis, which is an inflammatory disease (19–22). The NF‐κB pathway is involved in a wide range of biological activities, including cell inflammation, proliferation, apoptosis, and lymphangiogenesis (23). In addition, BST2 acts as a potent activator of NF-кB; it can mediate NF-кB activation through the YXY motif in its cytoplasmic domain, and interaction with TAK1 is required for NF-кB activation (19). Elucidating the mechanism underlying endometriosis may provide comprehensive insight to better prevent endometriosis formation and progression. In light of this background, the current study aimed to investigate the molecular mechanisms underlying lymphangiogenesis in endometriosis by which NF-κB signaling is activated by BST2.

## 2 Materials and methods

### 2.1 Human tissue collection

All the tissue samples were collected in the First Affiliated Hospital of Harbin Medical University according to protocols that were approved by the Ethics Committee of First Affiliated Hospital of Harbin Medical University, and the experiments were conducted following the World Medical Association Declaration of Helsinki. Written informed consent was obtained from each subject prior to harvesting tissue samples. In total, 40 women undergoing gynecologic laparoscopic surgery were recruited for the study. Endometriotic tissues were collected from the inner side of the cyst wall of endometriomas in patients with ovarian endometriosis, and these patients were diagnosed by laparoscopic surgery and histopathologic examination (n=20); normal endometrial tissues (confirmed by pathologic diagnosis) were obtained during total hysterectomies with uterine leiomyomas (n=20). All women with or without endometriosis had regular menstrual cycles. None of the patients had received steroid hormone treatment for at least 3 months before the surgical procedure. Patients with a history of hypertension, diabetes mellitus, acute infection, autoimmune disorder, and other significant diseases were excluded from this study. Tissue samples were snap-frozen in liquid nitrogen until further analysis or immediately processed for primary cell culture studies. An aliquot was fixed in 4% paraformaldehyde and embedded in paraffin for pathological diagnosis and immunohistochemical (IHC) assessment.

### 2.2 Isolation, culture and characterization of ESCs

Under sterile conditions, endometrial samples from endometriosis and nonendometriosis patients were collected and kept in Dulbecco’s modified Eagle’s medium/Ham’s F-12 medium (DMEM/F-12) supplemented with 10% fetal bovine serum (FBS) and 1% penicillin–streptomycin (100 U/mL penicillin and 100 ug/mL streptomycin) on ice. Within 6 h after collection, the tissues were washed with phosphate-buffered saline (PBS) several times to remove traces of blood, minced into small pieces of 1mm^3^ and digested with collagenase IV (1 mg/ml, Biosharp) for 60-90 min with shaking at 37 °C. Then, the normal endometrial stromal cells (NESCs) and ectopic endometrial stromal cells (EESCs) were successively filtered through sterile nylon mesh to remove the remaining debris and epithelial cells, and the cells were finally cultured in DMEM/F-12 medium supplemented with 10% FBS and 1% penicillin/streptomycin in a humidified atmosphere with 5% CO_2_ at 37°C. Vimentin (positive marker) and E-cadherin (negative control epithelial cell marker) antibodies were used to verify the particular characteristics of stromal cells by immunofluorescence. Only cells that were cultured with more than 95% purity and passaged every 2-3 days were included in our study. Human lymphatic endothelial cells (HLECs) were purchased from Promocell (Wuhan, China) and maintained in Dulbecco’s modified Eagle’s medium (DMEM) supplemented with 10% FBS and 1% penicillin/streptomycin at 37°C in a humidified incubator in 5% CO_2_.

### 2.3 Immunohistochemical staining

IHC staining was performed on paraffin-embedded tissue samples. Section slides with tissue were deparaffinized twice in xylene and rehydrated in twice absolute ethyl alcohol, followed by incubation with a graded series of ethanol solutions. Then, the slides were treated with antigen retrieval buffer and incubated with hydrogen peroxide at room temperature for 15 min to block endogenous peroxidase activity before incubation with the indicated primary antibody at 4°C overnight. On the second day, secondary antibodies were added and incubated at room temperature for 30 min, and peroxidase staining was then visualized with 3,3’-diaminobenzidine (DAB) substrate. Random fields of each section were photographed.

### 2.4 Cell transfection

RNA interference was performed by small interfering RNA (siRNA) transfection. SiRNAs against BST2 (Si-BST2) and a negative control siRNA (Si-NC) were designed and synthesized by GenePharma (Suzhou, China). Cell transfection was carried out using jetPRIME reagent (Polyplus, France) according to the manufacturer’s specifications. After incubation for 48 h, the cells were collected for subsequent experiments.

### 2.5 RNA extraction and quantitative real-time PCR

Total RNA was isolated from tissues or cells using TRIzol (Invitrogen, USA) according to the manufacturer’s instructions, and the concentrations and quality of RNA were determined by a spectrophotometer (NanoDrop, USA). Reverse transcription was performed using FastKing gDNA Dispelling RT SuperMix (Tiangen, Beijing, China). Then, the cDNA samples were used for real-time PCR and quantified with Talent qPCR PreMix (SYBR Green) (Tiangen, Beijing, China). We used glyceraldehyde 3-phosphate dehydrogenase (GAPDH) as the housekeeping gene, and the relative expression levels of mRNA were calculated by using the 2–^ΔΔCt^ or –ΔCt method. The specific primers that were used for amplification were synthesized by Ruibiotech (Beijing, China), and the sequences of the primers used for PCR are listed in **Supplementary Table S1**.

### 2.6 Protein extraction and western blotting analysis

Tissues or cells were washed twice in cold PBS prior to being lysed with radioimmunoprecipitation assay (RIPA) buffer (Beyotime Biotechnology, Shanghai, China) supplemented with protease inhibitors and phosphatase inhibitors according to the manufacturer’s instructions. Then, the protein concentrations were measured by the BCA method (BCA Protein Assay Kit, Tiangen, Beijing, China). Aliquots of equal protein amounts were loaded into 12.5% sodium dodecyl sulfate−polyacrylamide gel electrophoresis (SDS−PAGE) gels and electrophoretically transferred to polyvinylidene difluoride (PVDF) membranes (Roche, Mannheim, Germany). Then, the membranes were blocked with 5% fat-free milk for 1 h at room temperature and then incubated with the appropriate primary antibody overnight at 4°C. Detailed information about the antibodies is provided in **Supplementary Table S2**. On the following day, the membranes were washed with TBST and incubated with horseradish peroxidase (HRP)-conjugated secondary antibodies at room temperature for 1 h. The protein bands were developed using a BeyoECL Moon kit (Beyotime Biotechnology, Shanghai, China) for visualization according to the manufacturer’s instructions. Analysis of bands was performed using ImageJ software (Bethesda, MD, USA), and the data were normalized to the internal gene expression.

### 2.7 Immunofluorescence staining

Cells on glass coverslips were fixed for 30 min with 4% paraformaldehyde, permeabilized with 0.3% Triton X-100 in PBS wash buffer for 15 min, and blocked with 5% normal goat serum for 1 h at room temperature. The cells were then stained with the appropriate fluorescence-coupled primary and secondary antibodies. Finally, the cell nuclei were stained with 4,6-diamino-2-phenylindole (DAPI) (Beyotime Biotechnology, Shanghai, China). The cells were visualized under a fluorescence microscope (Olympus, Japan).

### 2.8 Cell counting Kit-8 assay

Cell proliferation assays were carried out using the Cell Counting Kit-8 (CCK-8) assay (MCE, NJ, USA) according to the manufacturer’s protocol. Transfected cells were plated in 96-well plates at a density of approximately 3000 cells in 90 μl of medium per well. Then, 10 μL of CCK-8 solution was added to each well, followed by incubation at 37°C for 2 h. The optical density (OD) value was measured at a wavelength of 450 nm by a microplate reader (Thermo Fisher Scientific, USA) every 24 h for 3 days.

### 2.9 5-Ethynyl-2′deoxyuridine assay

A total of 4 ×10^3^ treated EESCs were incubated in 6-well plates. For EdU staining, 10 µM EdU reagent (Beyotime Biotechnology, Shanghai, China) in culture medium was added to each well and incubated for 3 h at 37°C. Finally, the cells were stained with Hoechst 33342 according to the manufacturer’s protocol and observed under a fluorescence microscope. The cell proliferation rate was calculated as the proportion of EdU-positive cells (green dots) to total Hoechst 33342-positive cells (blue dots).

### 2.10 Wound healing assay

After incubation and reaching 90–100% confluence, the treated EESCs layer was scratched with a 200 µl sterilized pipette tip, and floating cells and debris were washed away with PBS. Wound healing was observed and photographed at 0 h, 24 h, and 48 h after scratching. Finally, the wound size at each time point was normalized to that at the 0 h time point, and the results are reported as the percent area closed.

### 2.11 Transwell migration assay

Cell migration assays were carried out in 24-well Transwell chambers (Corning, NY, USA). Eight-micrometer pore inserts were used, and cells (2 × 10^5^) in 200 μl of serum-free medium were added to the upper chamber. A total of 700 μl medium supplemented with 20% fetal bovine serum was added to the lower chamber as a chemoattractant. After 24 h in an incubator at 37°C, the migratory cells on the lower membrane surface were fixed, stained, and counted in random fields, after the cells inside the upper chamber were removed with cotton swabs.

### 2.12 Flow cytometry analysis

FCM was used to quantify the apoptosis rate of EESCs with an annexin V-fluorescein isothiocyanate (FITC)/propidium iodide (PI) staining assay kit (Beyotime Biotechnology, Shanghai, China) according to the manufacturer’s instructions. To analyze the apoptosis rate, 4×10^5^ EESCs were seeded in 6-well plates. After transfection for 48 h, the cells were stained and analyzed using flow cytometry (BD Biosciences, USA).

### 2.13 Enzyme-linked immunosorbent assay

The concentrations of human VEGFC in the cell culture supernatants were measured with a Human VEGFC ELISA Kit (CSB-E04759h, CUSABIO, Wuhan, China) according to the manufacturer’s instructions. Briefly, 96-well polystyrene microplates were coated with an antibody specific for human VEGFC. After treatment, the cell culture supernatants were added to the microplates and incubated at 37 °C for 2 h before sequentially adding the corresponding antibody, substrate solution, and stop solution. Finally, the optical density of each well was measured at 450 nm by using a microplate reader.

### 2.14 HLEC proliferation assay, migration assay and tube formation assay

EESCs were seeded in 6-cm dishes and transfected with siRNA. Forty-eight hours later, the cell culture media were collected and used for subsequent assays. For the proliferation assay, HLECs were cultured in 96-well plates with conditioned cell culture media. Then, 10 µl CCK-8 solution was added to the plates. After incubation for 2 h at 37°C, the optical density values were measured every 24 h for 3 days.

Wound healing assays and Transwell assays were also performed to evaluate the migration ability of HLECs after being cultured with conditioned medium according to the corresponding protocol.

For the tube formation assay, Matrigel (50 µl) was added to each well of a 96-well plate on ice and allowed to polymerize for 30 min at 37°C. A total of 2×10^4^ HLECs in 100 µl of conditioned medium were added to each well and incubated at 37°C in 5% CO2. After 6 h, the lymphatic tubes were photographed, and the total length and branches numbers of tubule structures were measured and quantified.

### 2.15 Dual-luciferase reporter gene assay

The 5’-promoter regions of BST2 were inserted into the pGL4.18-basic plasmid. The BST2 reporter plasmid, control-luciferase plasmid and pRL-TK Renilla plasmid were transfected into EESCs using transfection reagent according to the manufacturer’s instructions. After 24 h of transfection, firefly luciferase activity and Renilla luciferase activity were measured by using the Dual-Luciferase Reporter system (Promega, Wisconsin, USA), and the firefly luciferase activity was normalized to the Renilla luciferase activity.

### 2.16 Animal experiments

All the animal handling and experimental procedures were approved by the Animal Experimental Ethics Committee of the First Affiliated Hospital of Harbin Medical University. A total of 10 female BALB/c nude mice aged 4–6 weeks were purchased from Beijing Vital River Laboratory Animal Technology Limited Company and housed under a 12/12-h light-dark cycle, with standard chow and water were provided ad libitum. Endometrial stromal cells were collected under sterile conditions and subcutaneously injected into the axillae of nude mice. The lesions were allowed to grow for two weeks, and the mice were then randomly divided into two groups to receive either an intralesional injection of Si-BST2 or a negative control. The mice were sacrificed after one month of drug treatment, and the endometriotic-like lesions were excised and measured for subsequent experiments.

### 2.17 Statistical analysis

All the morphometric data were collected in a blinded manner. All the data were analyzed by GraphPad 7.0 software (GraphPad Software, USA), and the results are presented as the means ± standard errors of the means (SEM). Student’s t test was used for comparisons between the two groups, and one-way analysis of variance (ANOVA) followed by Tukey’s test was used for comparisons of multiple means. All the experiments were repeated at least 3 times using different batches of cells. P values of 0.05 or less were considered statistically significant for all tests (ns, p ≥ 0.05; *p < 0.05; **p < 0.01; ***p < 0.001; ****p < 0.0001).

## 3 Results

### 3.1 BST2 was overexpressed in endometriosis and positively correlated with lymphangiogenesis

**Figure 1:**
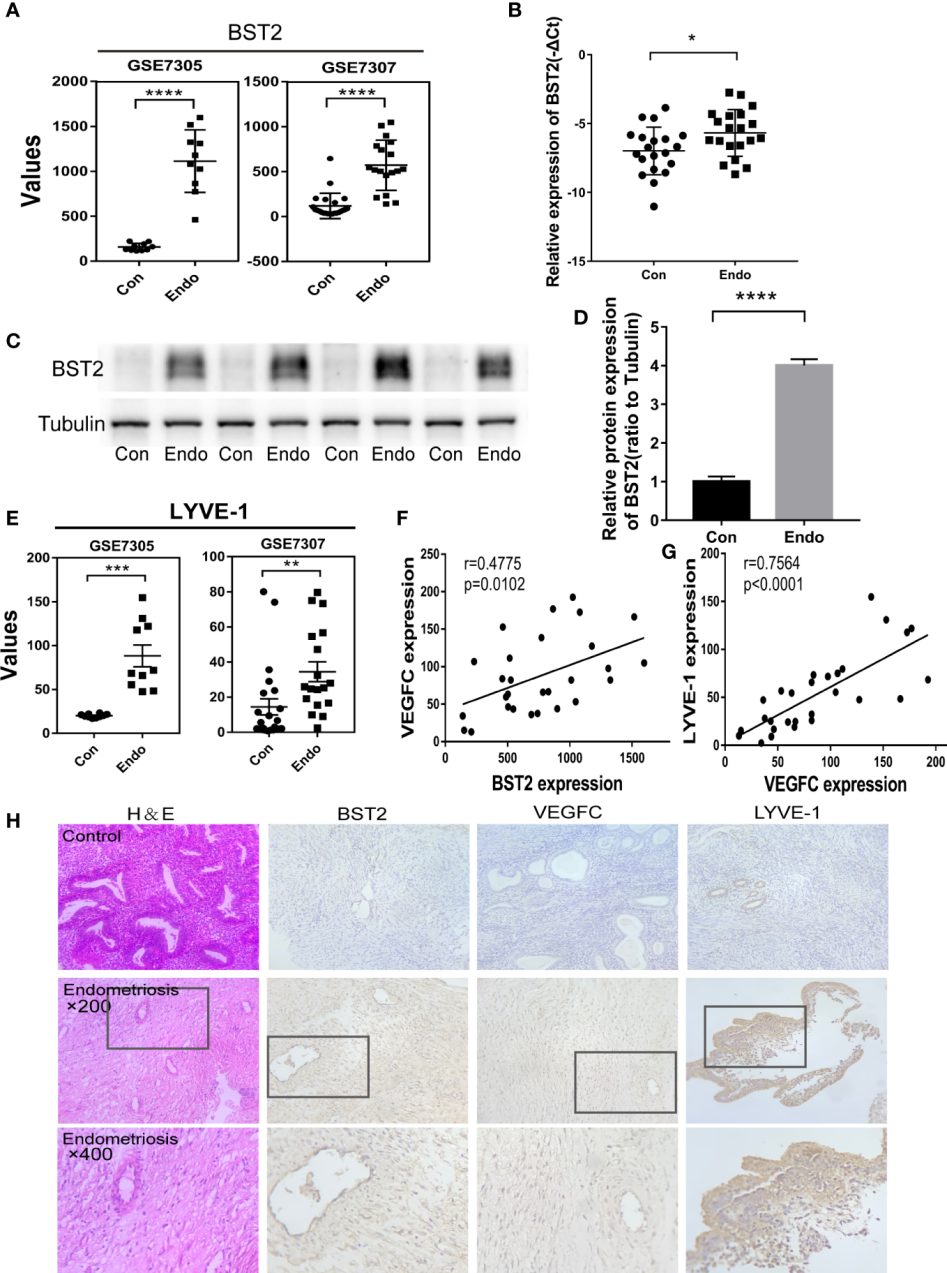
BST2 levels were overexpressed and positively correlated with the lymphangiogenesis in the endometriosis. **(A)**, BST2 expression levels in the GSE7305 and GSE7307 datasets. **(B–D)**, BST2 mRNA **(B)** and protein **(C, D)** expression in endometriosis samples. **(E)**, LYVE1 expression levels in the GSE7305 and GSE7307 datasets. **(F)**, The correlation of BST2 expression and VEGFC expression in the GSE7305 and GSE7307 datasets. **(G)**, The correlation of VEGFC expression and LYVE1 expression in the GSE7305 and GSE7307 datasets. **(H)**, **(H, E)** staining and Immunohistochemistry (IHC) of BST2, VEGFC and LYVE1 in endometriosis and control group. *p < 0.05; **p < 0.01; ***p < 0.001; ****p < 0.0001.

To systemically identify novel targets in EM, we analyzed our preliminary bioinformatics results, and we collected tissues from clinical subjects to further verify that BST2 was differentially expressed in EM and to explore its role in EM. Based on the GEO database, BST2 high expression was observed in the GSE7305 and GSE7307 datasets (**Figure 1A**). Consistent with the above results, we verified that BST2 was actually upregulated at both the RNA and protein levels in the endometriosis group of our collected samples (**Figures 1B–D**). Moreover, LYVE1, which was a widely used marker of lymphangiogenesis, was also highly expressed in the GSE7305 dataset and GSE7307 dataset (**Figure 1E**). We then investigated the strong correlation between lymphangiogenesis and the VEGFC, which was a pivotal point in the development of lymphangiogenesis, in the GSE7305 and GSE7307 datasets (**Figure 1F**). More importantly, the data showed a positive correlation between BST2 expression and VEGFC expression in ectopic lesions, which may indicate that BST2 had a positive effect on lymphangiogenesis in EM (**Figure 1G**). Additionally, the IHC images also directly showed the high expression of BST2, VEGFC, and LYVE1 in the EM group compared to the control cases (**Figure 1H**). Taken together, these results suggested that a novel dysregulated membrane protein, BST2, may be involved in EM lymphangiogenesis and is therefore a candidate gene for further investigation.

### 3.2 BST2 induced proliferation and inhibits apoptosis of EESCs *in vitro*

**Figure 2:**
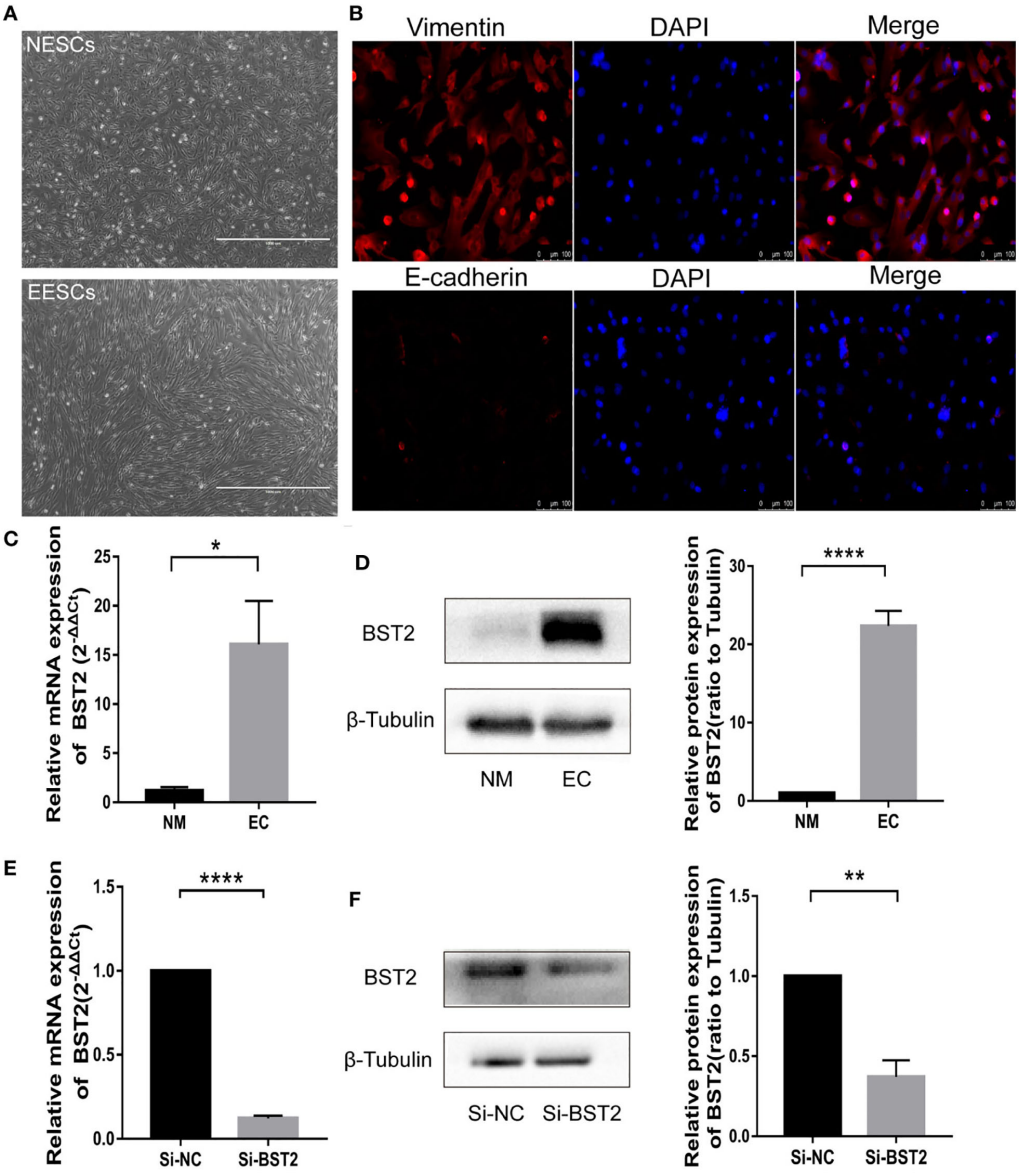
Identification of endometrial stromal cells and BST2 expression at the cellular level. **(A, B)**, The identification of endometrial stromal cells on the morphology **(A)** and Immunofluorescence **(B)**. **(C, D)**, BST2 mRNA **(C)** and protein **(D)** expression at the cellular level. **(E, F)**, Identification of the transfection efficiency of Si-BST2 at the mRNA **(E)** and protein **(F)** levels. *p < 0.05; **p < 0.01; ****p < 0.0001.

**Figure 3:**
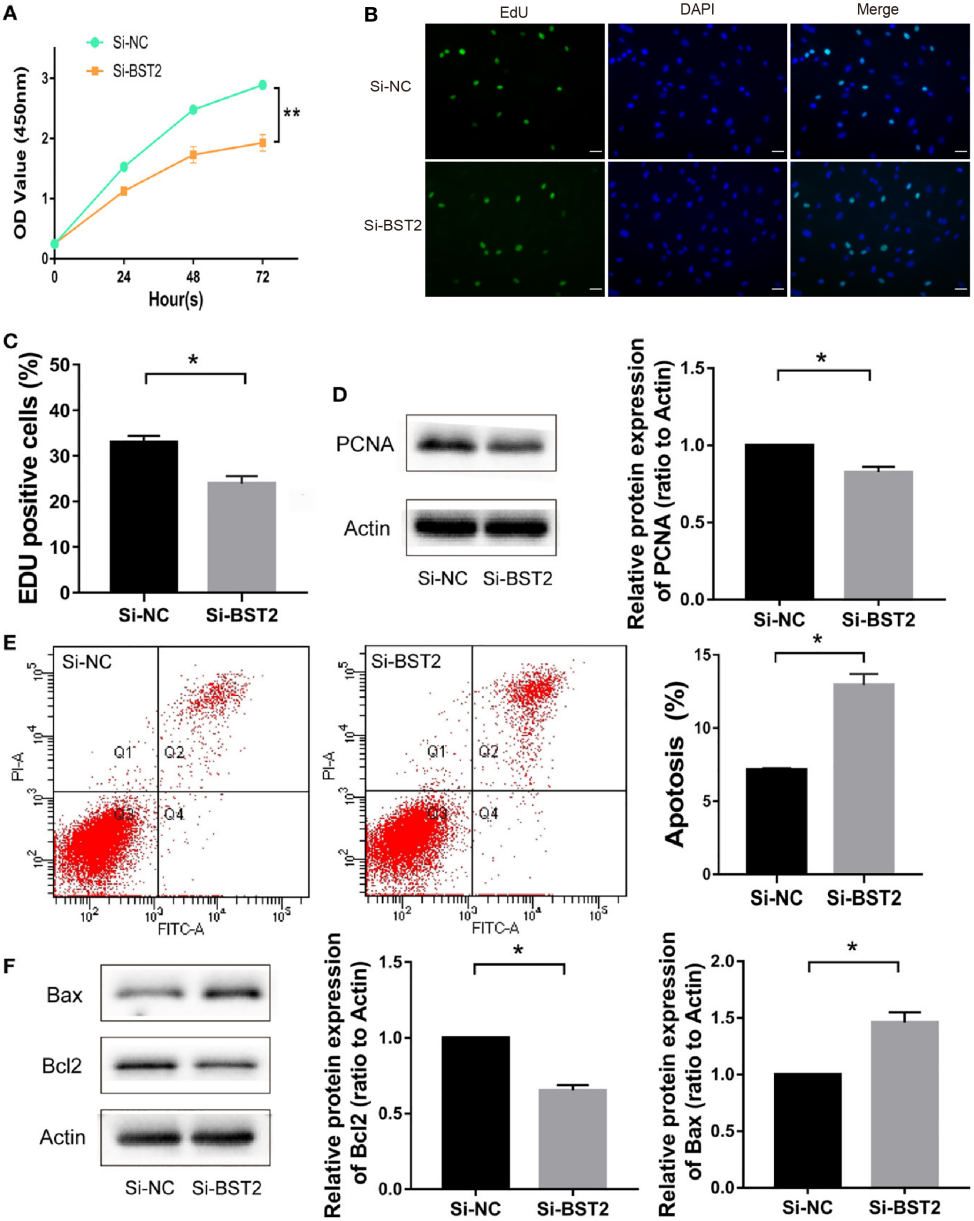
The effect of BST2 on cell proliferation and apoptosis. **(A–C)**, The proliferation of EESCs was examined by CCK-8 assay **(A)** and EdU **(B, C)** after Si-BST2 or Si-NC treatment for 48 h. **(D)**, The proliferation-related protein PCNA was measured by Western blot assay after Si-BST2 or Si-NC treatment for 48 h. **(E)**, The Flow cytometry was performed to detect the apoptosis rates of the EESCs transfected with Si-BST2 or Si-NC. **(F)**, Western blot analysis was used to measure the levels of Bax and Bcl2 in cells transfected with Si-BST2 or Si-NC. *p < 0.05; **p < 0.01.

To investigate the biological function of BST2 in EESCs, we first determined the cell purity based on morphology and cellular marker protein expression (**Figures 2A, B**), and then examined the expression of BST2 at both RNA and protein levels; EESCs exhibited higher levels of BST2 than NESCs (**Figures 2C, D**). Then, using BST2-targeting siRNA, we efficiently downregulated BST2 expression in EESCs (**Figures 2E, F**) and further explored whether BST2 is involved in cell proliferation and apoptosis. Cell proliferation was assessed by CCK-8 analysis and EdU assay. The CCK-8 results demonstrated that BST2 knockdown significantly reduced the proliferation of EESCs compared to control cells (**Figure 3A**). This was further illustrated by the EdU assays, as EdU-positive staining showed a reduction in the proliferation of EESCs after BST2 knockdown (**Figures 3B, C**). In addition, the expression level of the proliferation-related protein PCNA was correspondingly decreased after transfection with Si-BST2 (**Figure 3D**). Taken together, these findings indicated that BST2 promoted the proliferation of EESCs. Cell proliferation was partially regulated by apoptosis. To assess the impact of BST2 on apoptosis in EESCs, flow cytometry assays were performed. After transfection with BST2 siRNA, the EESCs exhibited higher levels of apoptosis than the corresponding control cells (**Figure 3E**). Furthermore, in cells transfected with Si-BST2 for 48 h, the protein expression level of Bax was notably elevated, whereas the expression of the Bcl2 was significantly decreased (**Figure 3F**). Thus, these results strongly suggested that BST2 may contribute to the progression of endometriosis *in vitro*.

### 3.3 Knockown of BST2 markedly suppressed the migration of EESCs *in vitro*

**Figure 4:**
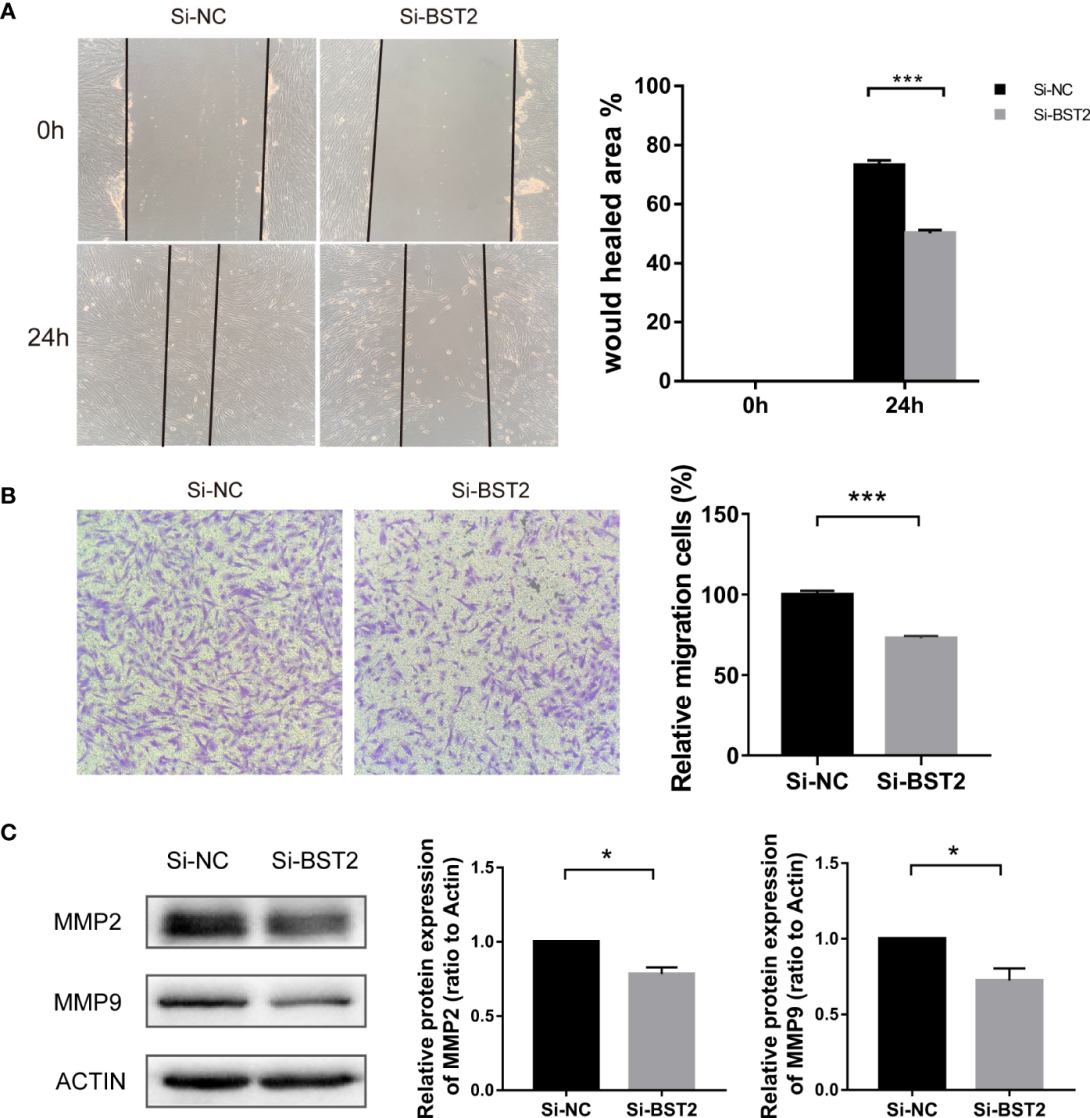
Effect of BST2 on cell migration. **(A)**, Representative images and quantification of cell motility changes in wound healing assays at 0h and 24h. **(B)**, Representative images and quantification of transwell migration assays at 24h after BST2 knockdown. **(C)**, The migration-related protein bands were performed by the western blot assay after transfection with Si-BST2 or Si-NC for 48h. *p < 0.05; ***p < 0.001.

Next, we investigated whether BST2 affected the migration of EESCs. We first confirmed the results with the scratch assay, and 24 h after scratching, the degree of wound healing was significantly decreased in the group transfected with BST2 small interfering RNA compared to the control group, suggesting that the migration of the cells was attenuated by the reduction in BST2 expression (**Figure 4A**). Similarly, this phenomenon was verified with a transwell migration experiment, in which the number of cells that migrated to the lower chamber was significantly decreased in the Si-BST2 transfected group compared to the Si-NC transfected group under the same chemotactic conditions (**Figure 4B**). Additionally, the expressions of MMP2 and MMP9, which were migration-related genes, were verified at the protein level, and the decrease in BST2 led to the diminished migration ability of EESCs (**Figure 4C**). Therefore, we concluded that BST2 has a positive regulatory effect on the migration of EESCs.

### 3.4 Knockdown of BST2 in EESCs inhibits lymphangiogenesis *in vitro*

**Figure 5:**
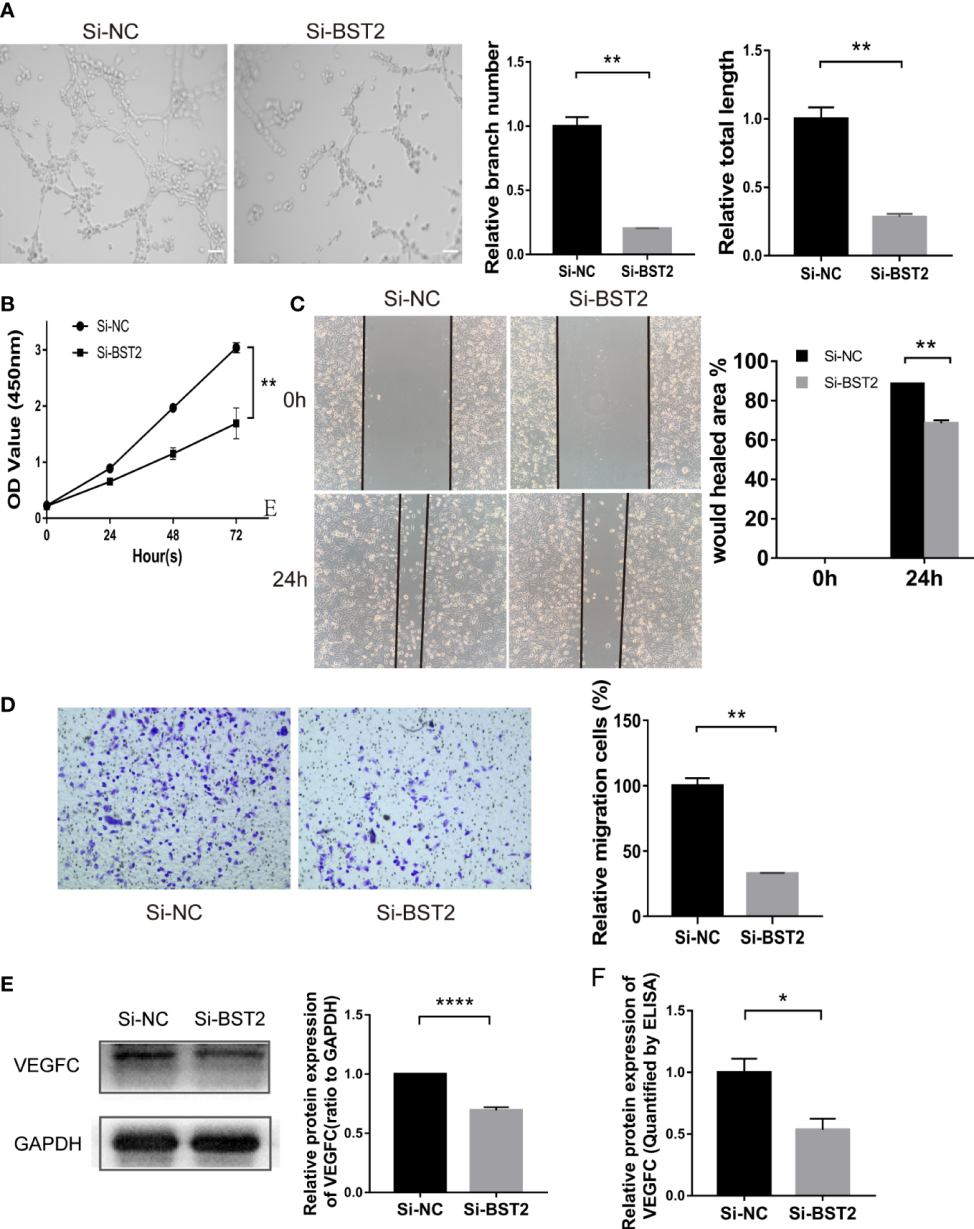
Effect of the conditioned medium (CM) of EESCs with BST2 knockdown on HLECs. **(A)**, The Tube formation assay was conducted to check the tube formation ability with the CM of EESCs with BST2 knockdown on HLECs. **(B)**, CCK-8 assay showing the proliferation of HLECs treated with CM from Si-BST2 or Si-NC EESCs for 24h. **(C, D)**, Scratch assay **(C)** and transwell assay **(D)** were conducted to assess migration ability of HLECs treated with CM from Si-BST2 or Si-NC EESCs for 24h. **(E)**, The VEGFC protein expression was measured by western blot analysis after transfection with Si-BST2 or Si-NC for 48h. **(F)**, The VEGFC expression from the conditioned medium (CM) of EESCs with BST2 knockdown was detected by ELISA assay. *p < 0.05; **p < 0.01; ****p < 0.0001.

Importantly, tube formation assays showed that the conditioned medium from BST2-silenced cells inhibited the ability of EESCs to induce HLEC tube formation, as the branch number and total length of the tubes were decreased compared with those of the control groups (**Figure 5A**). To determine the mechanism by which BST2 downregulation inhibited lymphangiogenesis, we conducted experiments to confirm whether conditioned media collected from BST2-knockdown EESCs affected HLECs. In the CCK-8 assay, the proliferation of HLECs could be significantly impaired by culture medium from BST2-knockdown cells compared with controls, indicating a crucial role of BST2 in lymphangiogenesis (**Figure 5B**). Additionally, a wound healing assay was performed to evaluate the motility of HLECs cultured with supernatants from BST2-knockdown or control cells (**Figure 5C**). Transwell migration assays revealed that the migratory rate of HLECs was dramatically decreased by the conditioned medium from BST2-silenced cells (**Figure 5D**). Therefore, our results suggested that the knockdown of BST2 reduced the proliferation and migration of HLECs and subsequently impaired the lymphangiogenesis of HLECs *in vitro*.

### 3.5 BST2 depletion reduced the expression and secretion of VEGFC in EESCs

VEGFC acted as a critical modulator of the progression of endometriosis by promoting lymphangiogenesis. Based on the bioinformatics outcomes described above showing a positive correlation between BST2 and VEGFC expression, we further performed a series of *in vitro* studies to validate VEGFC expression. First, VEGFC protein expression was measured by western blotting, and BST2 knockdown inhibited VEGFC expression in EESCs (**Figure 5E**). Importantly, VEGFC secretion levels were measured using a specific ELISA kit and it was shown that VEGFC secretion was also down-regulated by BST2 depletion (**Figure 5F**). ​Therefore, there was a strong reason to conclude that the presence of VEGFC in conditioned medium further promoted lymphangiogenesis in HLECs. In summary, BST2 may promote lymphangiogenesis *via* VEGFC signaling.

### 3.6 BST2 activated NF-κB signaling pathway

**Figure 6:**
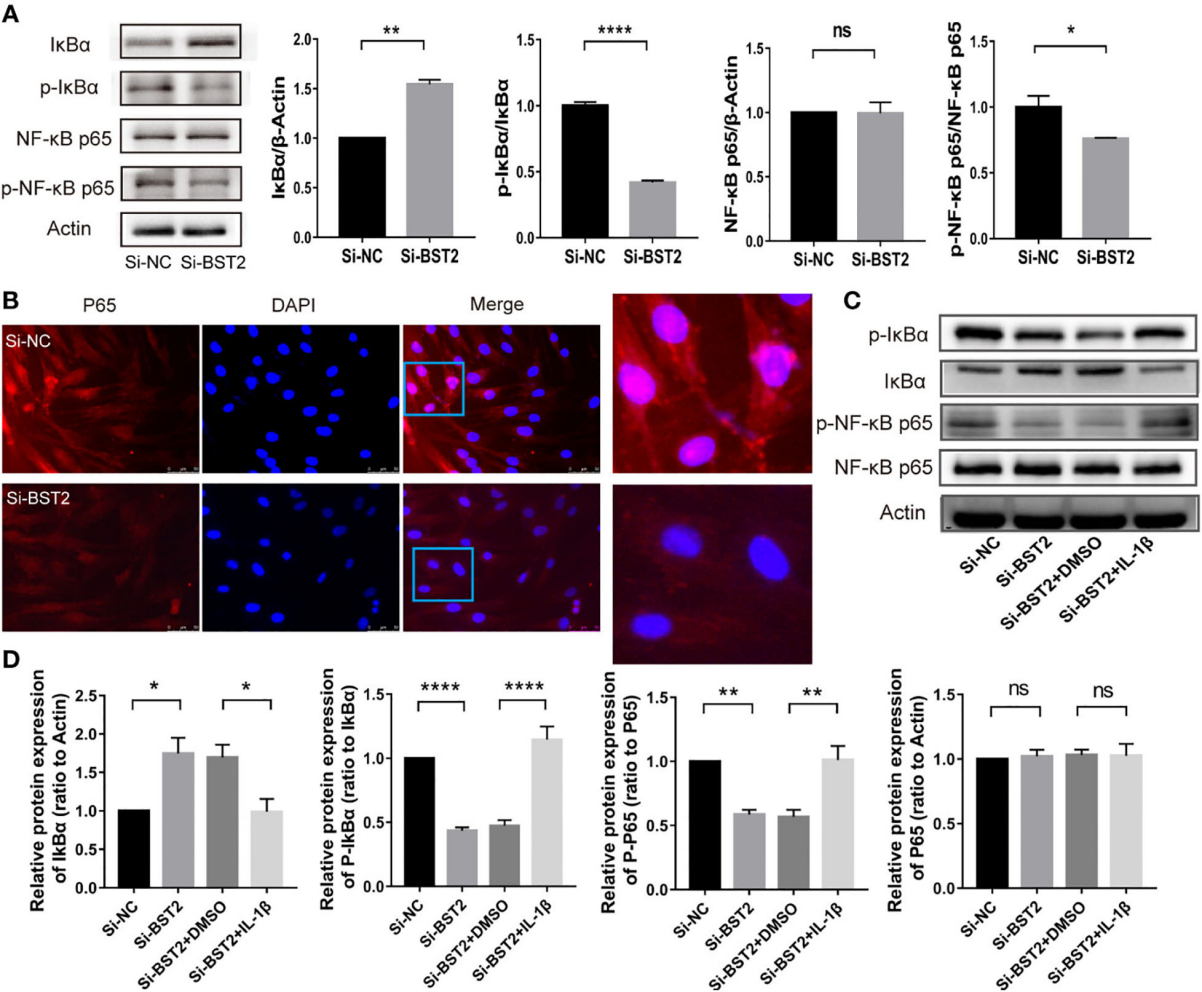
BST2 activated the NF-κB signaling pathway. **(A)**, The expressions of key proteins of NF-κB pathway were measured by western blot analysis after transfection with Si-BST2 or Si-NC for 48h. **(B)**, The Representative images of Immunofluorescence showed the cellular localization of NF-κB p65 protein in EESCs after transfection with Si-BST2 or Si-NC for 48h. **(C, D)**, The protein expressions of NF-κB pathway were measured by western blot analysis after treatment with Si-BST2 or Si-NC or/and the NF-κB pathway activator IL-1β. ns, p ≥ 0.05; *p < 0.05; **p < 0.01; ****p < 0.0001.

Previous studies reported that BST2 can activate NF-ҡB signaling (20, 24). To study the underlying mechanism in depth, we further assessed the effect of BST2 on NF-ҡB signaling in EESCs. We analyzed the expression of several key proteins in this signaling pathway by western blot assay (**Figure 6A**). Compared with the negative control, knockdown of BST2 expression reduced the phosphorylation of IκBα, which further led to the degradation of IκBα. Degradation of IκBα can lead to the nuclear translocation of various NF-κB complexes, predominantly the p50/p65 dimer. As expected, IF staining indicated a dramatic decrease in the nuclear translocation of NF-κB P65 when BST2 expression was knocked down (**Figure 6B**). Additionally, the NF-ҡB activator IL-1β was used to reverse the inhibitory effect of downregulated BST2 on NF‐κB pathway, as shown in **Figures 6C, D** (25, 26). After the addition of the agonist IL-1β, the phosphorylation levels of IκBα and p65 were significantly increased, observably reversing the reduction in phosphorylated IκBα and p65 caused by Si-BST2. These results suggested that BST2 may activate NF-κB signaling pathway.

### 3.7 BST2 regulated the progression of EESCs through the NF-κB signaling pathway

**Figure 7:**
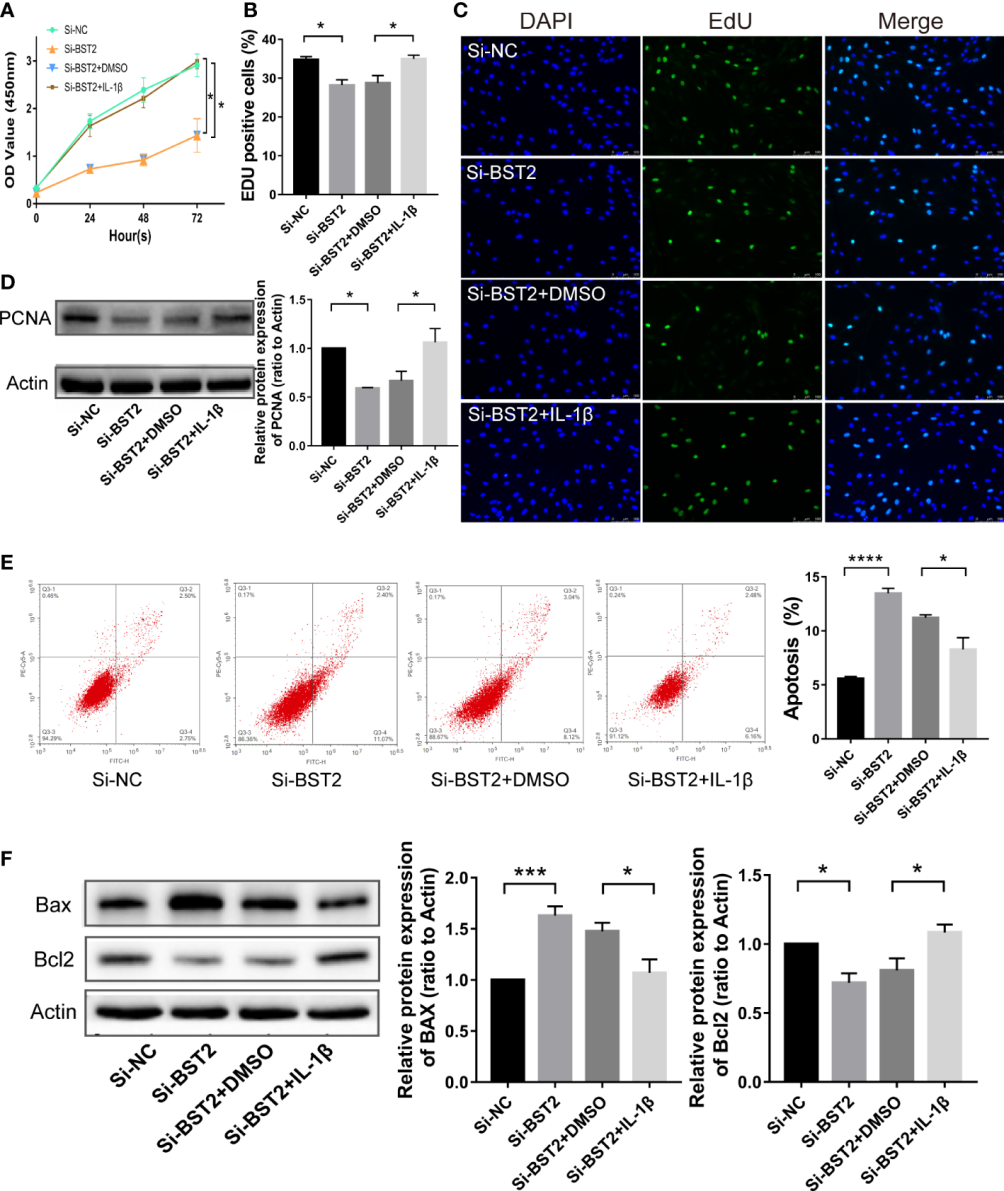
BST2 regulated proliferation and apoptosis of EESCs *via* the NF-κB signaling pathway. **(A–C)**, CCK-8 assay **(A)** and EdU staining assay **(B, C)** were conducted to measure the cell proliferation ability of EESCs after transfection with Si-BST2 and/or IL-1β. **(D, F)** The western blot analysis was used to examine the proliferation-related protein **(D)** and apoptosis-related protein **(F)** after transfection with Si-BST2 and/or IL-1β. **(E)**, The Flow cytometry was performed to detect the apoptosis rates of the EESCs transfected with Si-BST2 and/or IL-1β. *p < 0.05; ***p < 0.001; ****p < 0.0001.

**Figure 8:**
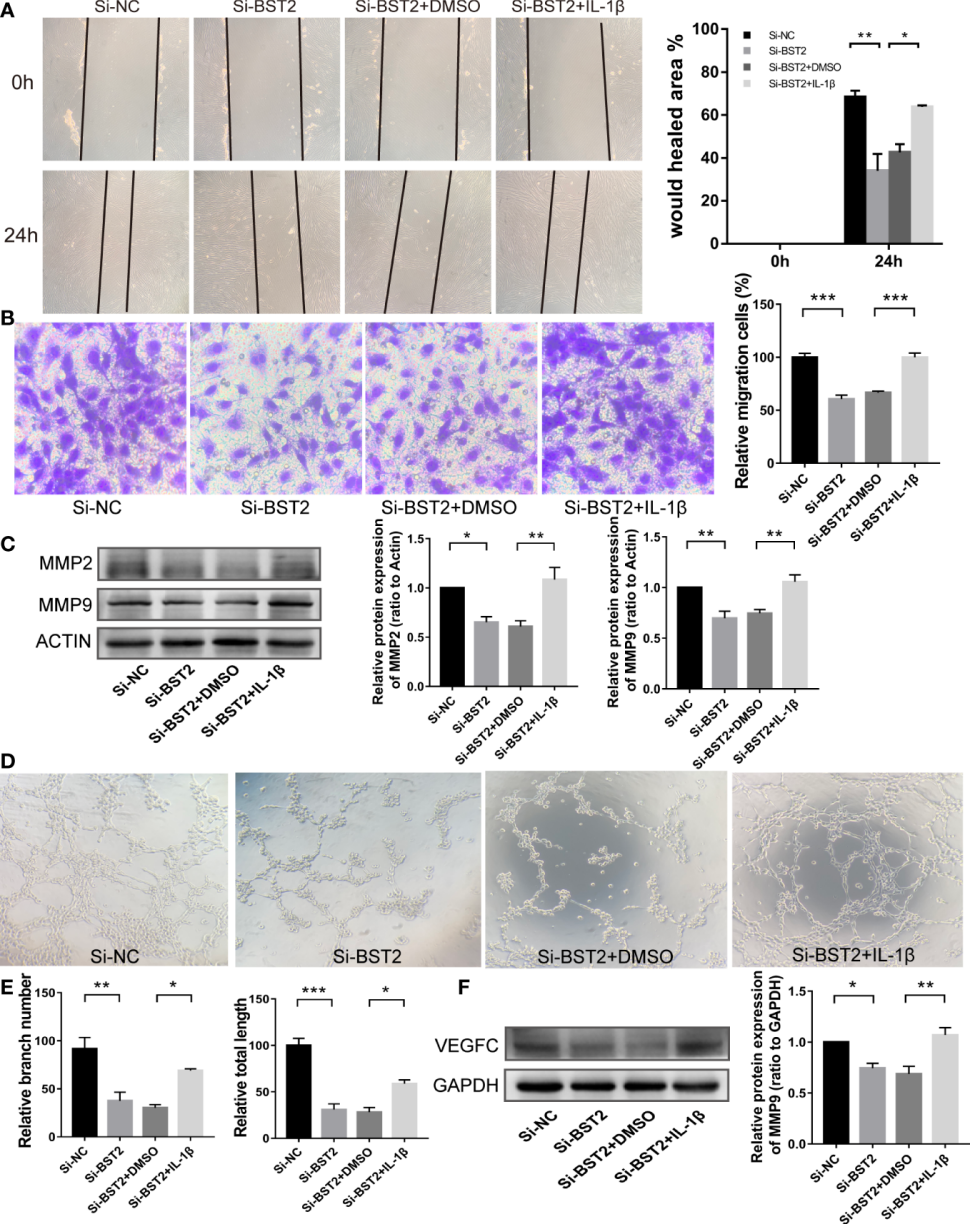
BST2 promoted migration and lymphangiogenesis of EESCs *via* the NF-κB signaling pathway. **(A, B)**, The wound healing assays **(A)** and the transwell assay **(B)** were performed to measure the cell migration ability of EESCs after transfection with Si-BST2 and/or IL-1β. **(C)**, The western blot analysis was used to examine the migration-related protein after transfection with Si-BST2 and/or IL-1β. **(D, E)**, The tube formation assay was conducted to check the tube formation ability with the CM of EESCs with Si-BST2 and/or IL-1β on HLECs. **(F)**, The western blot analysis was used to measure the VEGFC after transfection with Si-BST2 and/or IL-1β. *p < 0.05; **p < 0.01; ***p < 0.001.

Therefore, we hypothesized that BST2 regulates biological behaviour of EESCs *via* the NF-κB signaling pathway. To test this hypothesis, we assessed the bio-function of BST2-knockdown cells treated with or without the NF-ҡB activator IL-1β. IL-1β effectively rescued the effects of BST2 ablation on proliferation, as shown by the CCK-8 assay and EdU staining (**Figures 7A–C**). This finding was also supported by the results of PCNA, a proliferation-related protein measured by western blot assay (**Figure 7D**). Additionally, when Annexin V/PI double staining was used to analyze apoptotic cells that were treated with IL-1β, the cell apoptosis rate was notably rescued compared to the control group (**Figure 7E**), and the trends in the expression of apoptosis-related proteins were also consistent (**Figure 7F**). Similar results were also observed in the scratch assay and transwell chamber experiment, where IL-1β markedly reversed the Si-BST2-induced decrease in migration (**Figures 8A, B**). And the expression trends of migration-associated proteins were also in line with the above results (**Figure 8C**). These results strongly indicated that the induction of EESCs proliferation, migration and apoptosis by BST2 was most likely mediated through the NF-κB signaling pathway.

### 3.8 BST2 induced lymphangiogenesis and VEGFC high expression through the NF-κB signaling pathway

Accumulating evidence has shown that VEGFC signaling, which played a critical role in lymphangiogenesis, and p65, which was a transcription factor, could directly bind to the VEGFC promoter to enhance lymphangiogenesis (27). In addition, p65 could also bind to the promoter of the anti-apoptotic protein Bcl2, which was consistent with the results of our apoptosis results above. In the tube formation assay, after treatment with IL-1β, the branch number and total length of the tubes in the treatment group were rescued (**Figures 8D, E**). We next tested whether the expression of VEGFC on Si-BST2-transfected EESCs could be reversed with IL-1β treatment. As expected, the expression of VEGFC was partially increased (**Figure 8F**). Based on these outcomes, we can infer that BST2 can regulate the transcription of VEGFC *via* the NF-κB signaling pathway, thereby promoting lymphangiogenesis in endometriosis.

### 3.9 Elevation of BST2 in EESCs was mediated by the transcription factor IRF6

**Figure 9:**
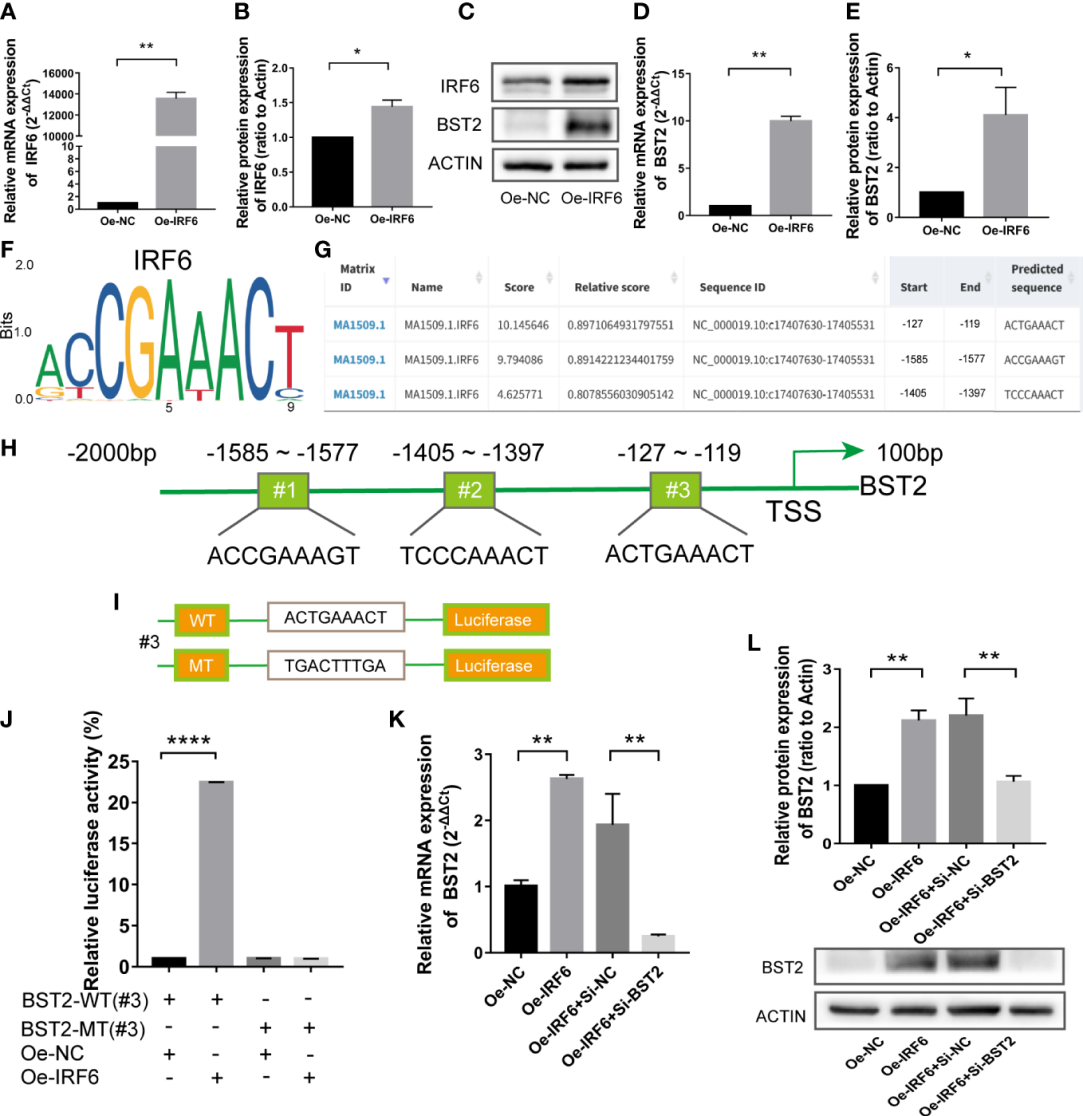
The transcription factor IRF6 directly binds to the promoter of BST2. **(A, B)**, The identification of the transfection efficiency of Oe-IRF6 at the mRNA **(A)** and protein **(B)** levels. **(C–E)**, The BST2 expression by the qRT-PCR **(D)** and western blot assay **(C, E)** after transfection with Oe-IRF6 for 48h. **(F)**, The IRF6 binding motif. **(G)**, JASPAR predicted the binding sites of IRF6 and BST2. **(H)**, The illustrative model of the binding sites of IRF6 and BST2. **(I)**, The illustrative model of the wild or mutant binding sites. **(J)**, The dual-luciferase reporter assay was performed to examine the transcriptional activity of the BST2 promoter. **(K, L)**, The expression of BST2 were detected by the qRT-PCR **(K)** and western blot experiment **(L)** after overexpression IRF6 and/or knockdown BST2. *p < 0.05; **p < 0.01; ****p < 0.0001.

Transcription factors (TFs) recognize specific DNA sequences to control chromatin and transcription, forming a complex system that guides the expression of the genome (28). To further elucidate the mechanisms underlying BST2 upregulation, we pay more attention on the TFs regulation. Combining our previous bioinformatic findings, we examined the transcription factor IRF6 to explore whether it regulated BST2 expression. We used an overexpression plasmid to alter IRF6 expression in EESCs, and the results showed that IRF6 overexpression markedly increased BST2 mRNA and protein expression in EESCs (**Figures 9A–E**). Subsequently, to identify potential IRF6 binding sites, we inspected the sequence of the BST2 promoter region using the JASPAR software and found three putative IRF6 binding sequences in the BST2 promoter region; among these sequences, sequence #3 had the highest score (**Figures 9F–H**). To verify that this potential IRF6-binding site was indeed responsive to IRF6, luciferase reporter plasmids carrying wild-type (WT) or mutant-type (MT) sequence #3 were generated and transfected into EESCs with or without IRF6 overexpression plasmid (**Figure 9I**). And the luciferase activity of the WT plasmid was dramatically increased in the IRF6-overexpressing group, and the increase disappeared when the binding site was mutated (**Figure 9J**). To further validate the association between IRF6 and BST2, qRT-PCR and western blot analysis were performed to identify the corresponding genes expression at the mRNA and protein levels (**Figures 9K, L**). Overall, IRF6 positively interacted with the BST2 promoter to promote BST2 transcription and further affected BST2 expression.

### 3.10 BST2 reversed IRF6-mediated proliferation, migration, apoptosis and lymphangiogenesis *in vitro*

**Figure 10:**
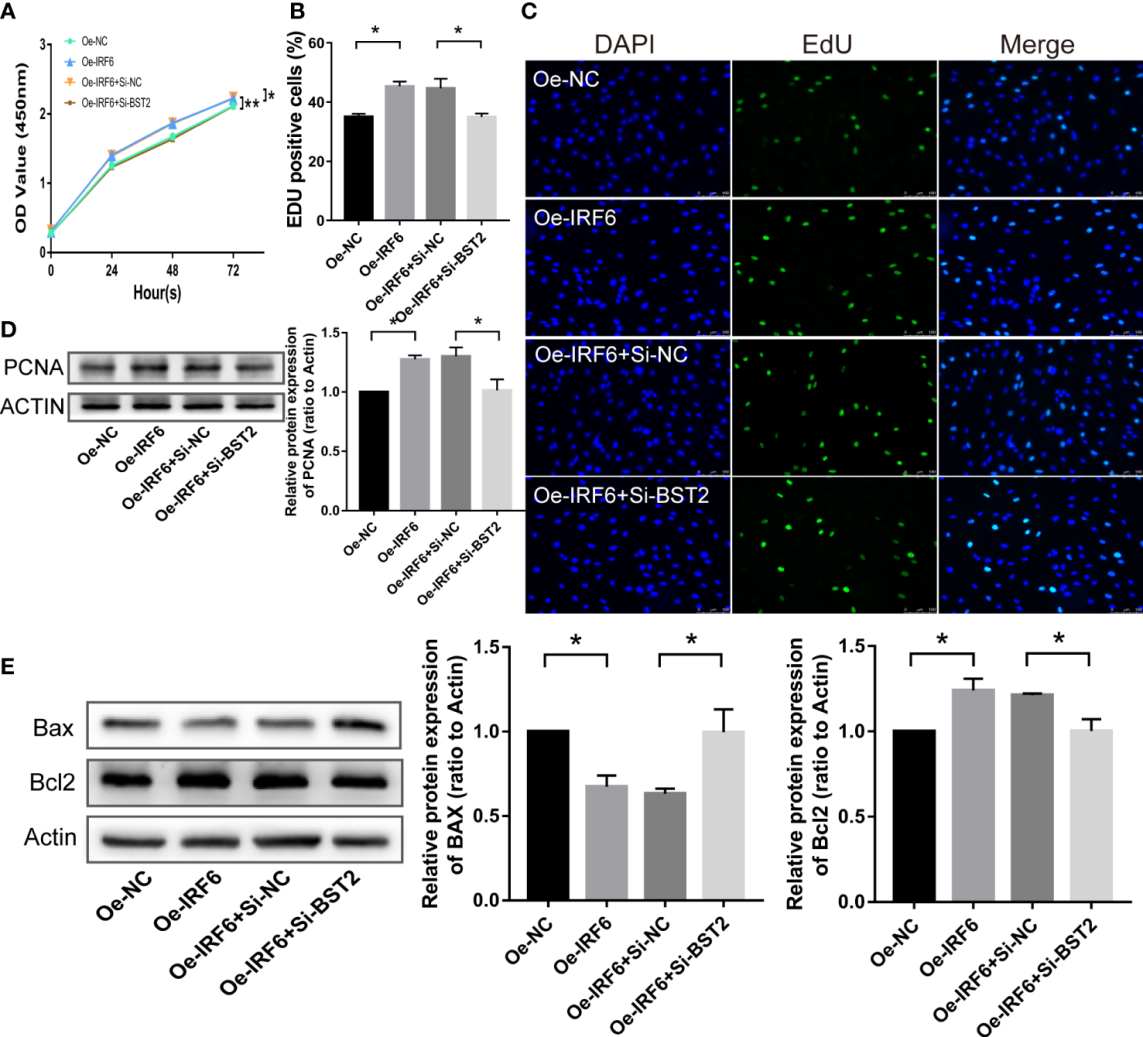
BST2 partially reversed the effects of IRF6 on the proliferation and apoptosis in EESCs. **(A–C)**, CCK-8 assay **(A)** and EdU staining assay **(B, C)** measured the cell proliferation ability of EESCs after transfection with Oe-IRF6 and/or Si-BST2. **(D, E)**, The western blot analysis examined the proliferation-related protein **(D)** and apoptosis-related proteins **(E)** after transfection with Oe-IRF6 and/or Si-BST2. *p < 0.05; **p < 0.01.

**Figure 11:**
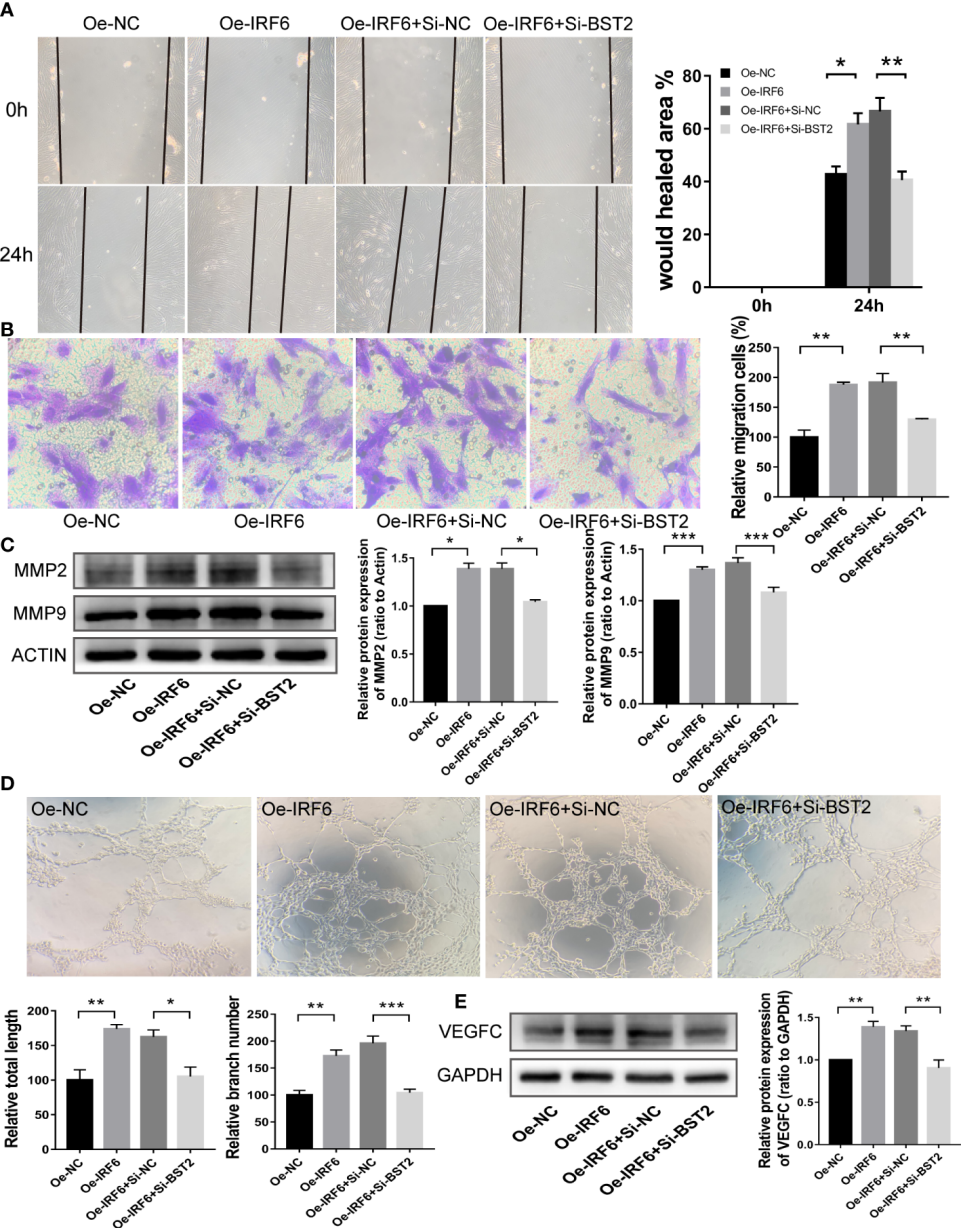
BST2 partially rescued the effects of IRF6 on the migration and lymphangiogenesis in EESCs. **(A, B)**, Wound healing assay **(A)** and Transwell assay **(B)** detected the cell migration ability after transfection with Oe-IRF6 and/or Si-BST2 for 48h. **(C)**, The western blot analysis examined the migration-related proteins after transfection with Oe-IRF6 and/or Si-BST2 for 48h. **(D)**, The tube formation assay checked the tube formation ability with the CM of EESCs transfected with Oe-IRF6 and/or Si-BST2 on HLECs. **(E)**, The western blot analysis measured the VEGFC after transfection with Oe-IRF6 and/or Si-BST2. *p < 0.05; **p < 0.01; ***p < 0.001.

Since an interaction between IRF6 and BST2 was determined, we further investigated whether IRF6 overexpression−induced cell biological behaviors were rescued by BST2 knockdown. The effect of IRF6 overexpression on promoting proliferation was abolished by BST2 knockdown, as shown by CCK-8 assay, EdU staining analysis and western blot assay (**Figures 10A–D**). Similar trends were observed in subsequent apoptosis rescue experiments. BST2 knockdown partially reversed the change in apoptotic-related proteins that had been caused by IRF6 overexpression (**Figure 10E**). Similar findings were also obtained in the migration experiments, which illustrated that overexpression of IRF6 could significantly promote the migration of EESCs, and reducing the expression level of BST2 could partially reverse this increase in migration (**Figures 11A–C**). Alter IRF6 overexpression, notably more tubes were observed in HLECs, but this number decreased after BST2 knockdown, as shown by the results of the tube formation assay (**Figures 11D–E**).

### 3.11 BST2 promoted proliferation and lymphangiogenesis *in vivo*

**Figure 12:**
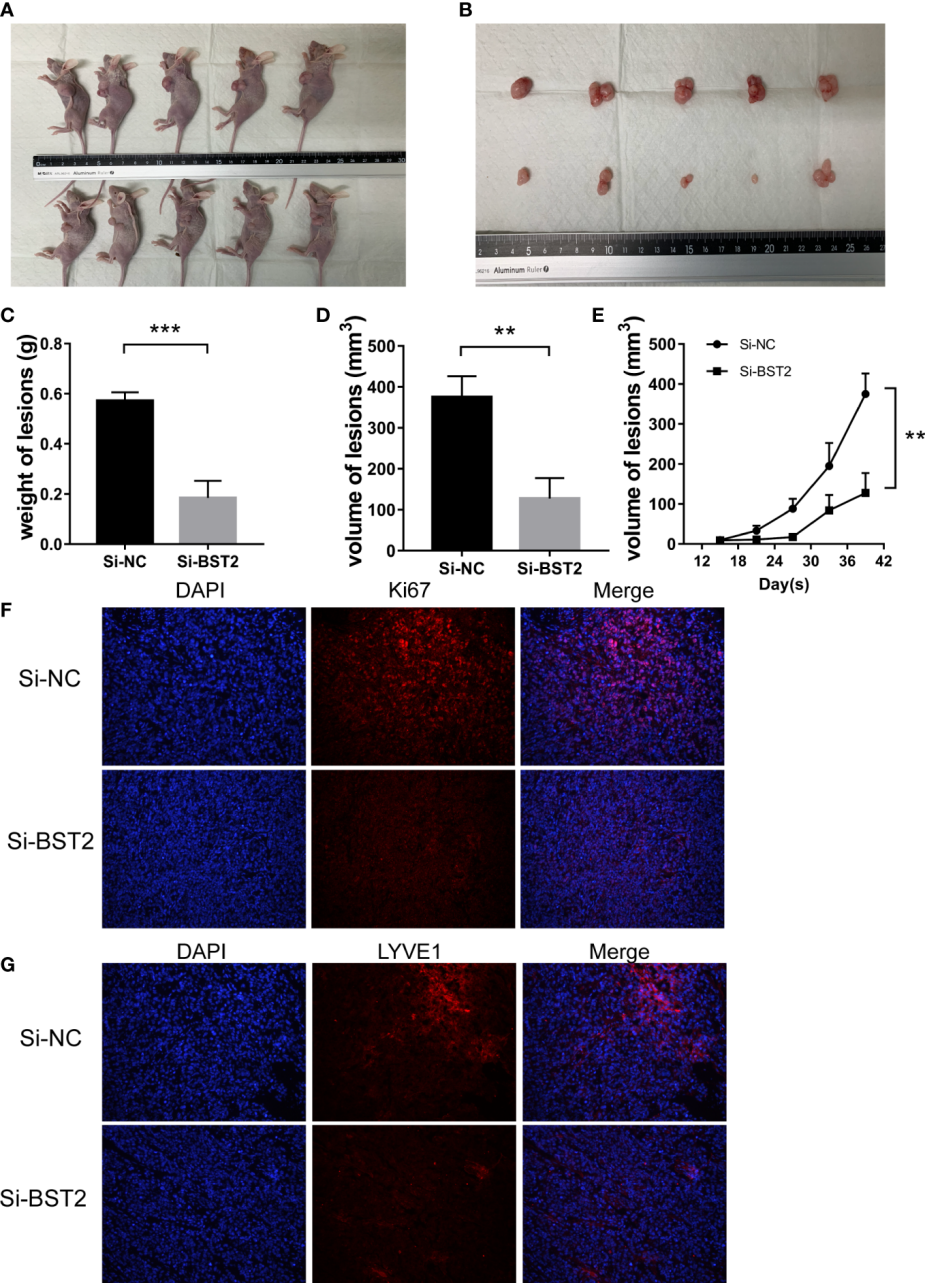
BST2 promoted the growth and lymphangiogenesis *in vivo*. **(A, B)**, The representative images of female BALB/c nude mice after subcutaneous injection with Si-BST2 and Si-NC. **(C, D)**, The weight **(C)** and volume **(D)** of lesion. **(E)**, The volume growth curves between the Si-BST2 group and Si-NC group. **(F, G)**, The representative images of Immunofluorescence showing the proliferation and lymphangiogenesis markers. **p < 0.01; ***p < 0.001.

To determine the effect of BST2 on the development of endometriosis lesions, we conducted further experiments to validate the characteristics of BST2 using a mouse model. The xenograft model demonstrated that the volume and weight of endometriosis in the BST2-knockdown group (n=5) was significantly lower than in the control group (n=5) (**Figures 12A–D**). In addition, the growth rate of endometriotic lesions in the transfected Si-BST2 group was decelerated (**Figure 12E**). Consistently, the IF results revealed that lesions derived from Si-BST2-transfected cells exhibited lower Ki-67 staining than lesions derived from control cells (**Figure 12F**). Moreover, IF staining also showed fewer lymphatic vessels (stained by LYVE1 antibody) in the Si-BST2-treated group (**Figure 12G**). Collectively, BST2 contributes to endometriosis lesion growth and lymphangiogenesis *in vivo*.

## 4 Discussion

Accumulating evidence has shown that a number of factors are involved in the onset and development of endometriosis; among them, immunity has attracted more attention in recent years. Thus, we have attempted to identify novel targets for drug therapies that may be involved in the immunological activity associated with endometriosis. Based on comprehensive biological information, BST2, an immune-related gene from the IMMPORT database, was selected for further analysis due to its substantial impact on other diseases. BST2, which is a host restriction factor, is often involved in the innate immune response and virus life cycle (29). In addition, BST2 also plays some roles in the progression of cancer. During the development of cervical cancer, BST2 induced cervical cancer cell growth, suppressed apoptosis and induced M1 macrophage but not M2 macrophage polarization (30). In gastric cancer, BST2 exerted oncogenic effects by regulating proliferation, apoptosis, and migration. BST2 was also a target gene of miRNA 760 and positively related to the progression of gastric cancer (31, 32). However, its role in the pathogenesis of endometriosis remains unknown.

In our study, we found that BST2 is aberrantly expressed in ectopic endometrial tissue, and that this up-regulated BST2 is positively correlated with the expression of VEGFC, which is a definitive factor in the formation of new lymphatic vessels. Moreover, an increase in BST2 was also observed in EESCs compared to control cells, suggesting that BST2 may be involved in the development and pathogenesis of endometriosis. However, the mechanisms underlying the high expression of BST2 and the pathology function and signaling cascade of BST2 need further in-depth exploration. To the best of our knowledge, this is the first report that clearly delineates the regulation, signaling, biological processes, and therapeutic potential of BST2 in endometriosis.

To investigate the potential biological behavior of BST2 in endometriosis, we took advantage of a series of papers in PubMed. Ectopic endometrial stromal cells migrate outside the uterus for some reason, and they undergo cellular survival, proliferation, anti-apoptotic and lymphangiogenic processes to form and gradually increase lesions until these lesions can be recognized and treated. From a functional point of view, our results indeed suggest that BST2 can regulate cell proliferation, migration, and apoptosis in a cell culture system, as well as partially in a mouse model of endometriosis. ​In addition, newly formed and distinct abnormal lymphatic vessels that develop as a result of endometriosis lesion lymphangiogenesis may facilitate the spread of ectopic cells into systemic lumen structures and are associated with local invasion and distant metastasis. Combined with our *in vitro* and *in vivo* results, we have strong reasons to conclude that BST2 generates favorable conditions for the progression of endometriosis.

Based on our previous studies and the JASPAR software analysis, we hypothesize that the transcription factor IRF6 may regulate BST2 expression in endometriosis by directly binding to its promoter region (17). Interferon-regulatory factors (IRFs) family consists of nine members in mammals that generally possessed a novel helix-turn-helix DNA-binding structural domain, with the transcription factor IRF6, a member of the IRF family, being reported to play an essential role in the regulation of immunity and oncogenesis (33). IRF6 is involved in a wide variety of essential biological processes and has been proven to be important in cell growth, differentiation and apoptosis. IRF6 can target KIF20A and is further involved in key cellular functions including cell proliferation, invasion, migration and apoptosis (34). Using primary cultured EESCs as a model, the results from luciferase assay suggests that IRF6 is definitely involved in the changes in BST2 transcription and expression. As for the activation of IRF6 in disease, we retrieved the literatures and found that the following aspects can influence its activation. First, in terms of transcription factors, the transcription factor ZEB1 can negatively regulate the expression of IRF6 in gastric cancer, while the transcription factor ELF3 can positively activate IRF6 (35). Next, with regard to proteins, the basal layer enriched protein RACK1 prevented premature differentiation by repressing the expression of the transcription factor IRF6 (36). Subsequently, among non-coding RNAs, Linc SNHG14 was found to activate the IRF6 effect in glioma glucose metabolism by affecting the degradation of IRF6 mRNA and miR-221-5p could contribute to ischemic acute kidney injury by activating IRF6-mediated apoptosis (37, 38). Furthermore, IRF6 served as a downstream target gene of the NOTCH pathway in epidermal development, and activation of this pathway could further facilitate what IRF6 did in it (39). Strikingly, in the case of DNA methylation, DNA methylation of the CpG island in the promoter region had a negative impact on the expression of IRF6 and the methylation status of IRF6 is potentially associated with the sensitivity of melanoma to interferon (40). Hence, in the follow-up study, we will continue to investigate the activation patterns of IFR6 in endometriosis.

Although the intracellular signaling of BST2 is still unclear, further understanding of the mechanisms underlying the functions regulated by BST2 may shed light on novel therapeutic targets for endometriosis. Obviously, some inflammation factors, cytokines, and signaling pathways are strongly associated with endometriosis, which is an inflammatory disease. The NF-κB signaling pathway, which is a classical inflammatory pathway, influences a broad range of biological processes, including innate and adaptive immunity, inflammation, stress responses, B-cell development, and lymphoid organogenesis (41). It has been demonstrated that the NF-κB pathway is strongly associated with several critical regulators of the development of endometriosis. The NF-κB pathway is responsible for influencing the occurrence and progression of endometriosis by regulating the activity of ectopic endometrial cells (42). The abnormal viability of ectopic endometrial cells is linked to the secretion of the pro-inflammatory cytokine IL-8 mediated as well as the activation of the anti-apoptotic proteins Bcl2 and Bcl-xL by the activated NF-κB pathway (43–45). In animal models of endometriosis, knockdown of NF-κB expression significantly reduced PCNA production and microvessel density in ectopic lesions, suggesting that NF-κB could be considered as a therapeutic target for preventing ectopic lesion growth and angiogenesis (46). Aberrant adhesion of ectopic endometrial cells is a pivotal element in establishing endometriosis. Activated NF-κB signaling pathway can induce high expression of essential adhesion factors including CD44, ICAM-1 and VCAM1, and inhibition of NF-κB pathway can effectively reduce the adhesion capacity of ectopic endometrial cells (47, 48).

It is well recognized that the binding of IκBα, which is a cellular NF-κB inhibitor, to NF-κB in the cytoplasm strongly inhibits the activation of the NF-κB signaling pathway; then, IκBα is degraded after its phosphorylation and ubiquitination, resulting in free NF-κB that translocates into the nucleus to induce the transcription of its target genes (49). Given the role of BST2 in innate immunity, including its role in NF-кB activation and the subsequent transcription of NF-кB-dependent genes, we hypothesized that BST2 is an upstream regulatory molecule of NF-κB signaling (19, 50). Our results showed that BST2 phosphorylates IκBα and subsequently promotes its ubiquitination. Moreover, the levels of the transcription factor P65 in the nucleus were significantly increased and the corresponding target gene, VEGFC, was upregulated compared to those in the BST2 knockdown group. This provides favorable evidence for lymphangiogenesis in endometriosis at the molecular level. In addition, the increased expression of Bcl2, which is a downstream target gene of P65, was also closely associated with the anti-apoptotic properties of EESCs. Interestingly, the NF-κB pathway activator IL-1β partially reversed these outcomes after transfection with Si-BST2. In summary, our studies showed that BST2-induced NF-κB activation played a vital role in the development and progression of endometriosis.

Therefore, these results provide novel insights into targeted therapy for endometriosis, which we can attempt by reducing the amount of BST2 binding to NF-κB or by weakening the binding ability of BST2 to NF-κB. To begin with the number of bindings, we can implement the low expression of BST2 by the corresponding biological techniques. When the expression level of BST2 is reduced, it means that the binding to NF-κB pathway is decreased, which in turn weakens the activation of this pathway and lessen the effect of NF-κB on the biological behavior of endometriosis. In addition, it has been proposed that the YxY sequence in the cytoplasmic domain of BST2 is required for the induction of NF-κB, which is well conserved in placental mammals. The induction of NF-κB by BST2 is impaired by inhibition of TAK1 and the TAK1-associated pseudophosphatase TAB1; these interactions require the YxY sequence in BST2 (19). This may alert us that we can influence the activation of NF-kB and the development of endometriosis by the alteration of the crucial YxY sequence.

Although endometriosis is a kind of benign disease, previous reports suggest that it shares many similar features with cancers, such as abnormal cell migration, invasion, and unrestrained growth (51). On the basis of abnormal adhesion of ectopic endometrial cells, the heightened migratory and invasive capacity of ectopic endometrial cells is thought to be the main cause of adhesion and extension of ectopic lesions (52). Matrix metalloproteinases, a class of active protein hydrolases responsible for extracellular matrix degradation and dependent on zinc and calcium ions, are an instrumental enzyme family in modulating the dynamic balance of the extracellular matrix (53). In recent years, it has been found that the invasive capacity of endometrial cells is realized through the degradation of extracellular matrix by MMPs, and the amount of gene expression of MMPs is positively correlated with the invasive capacity of ectopic endometrium, therefore, the involvement of matrix metalloproteinases is decisive for the formation of ectopic lesion (54). As representative matrix metalloproteinases, MMP2 and MMP9 also serve as downstream target genes of NF-κB, and when the NF-κB pathway is activated, the expression of MMP2 and MMP9 is increased, and in turn, the migration and invasion of ectopic endometrial cells is enhanced, which is consistent with our above findings (53, 55–57).

A previous study indicated that the newly developed lymphatic system not only serves as a channel to remove the wastes from endometriotic cells but also provides a network that allows immune cells to reach the endometriotic lesions. The outcomes from that paper were that some immune cells, including F4/80- and CD11c-positive macrophages, granzyme B-positive NK cells/cytotoxic T lymphocytes, and IL-17–positive Th-17 cells, contribute to the pathogenesis of endometriosis by increasing the secretion of proinflammatory cytokines (58). Dysfunction in macrophage-mediated phagocytosis of aberrant cells that undergo retrograde transport to the peritoneal cavity is considered an important factor in the development of endometriosis. infiltrated macrophages fail to mount an efficient phagocytic response, thus allowing the implantation and propagation of endometrial tissues ectopically. Prostaglandin E2 can facilitate the formation of endometriosis lesions by suppressing the phagocytosis of macrophages (59–61). In addition, the abundance of neutrophils and macrophages in the peritoneal fluid of patients with endometriosis raises the level of vascular endothelial growth factor and triggers the progression of endometriosis (62). It has been reported that the cytotoxicity of NK cells was observed to be reduced in peritoneal cells from endometriosis patients, implying that the defective cytotoxic function of NK cells prevented the elimination of ectopic endometrial cells by those and led to the formation of endometriotic lesions (63). There is a finding that Th17 cells are existed in endometriosis and IL-17A, as a key effector molecule of Th17 cells, it can play a vital role in promoting the progression of endometriosis by inducing the secretion of pro-inflammatory factor IL-8 together with the proliferation of endometrial stromal cell and the expression of COX2 (64). Treg cells suppress a series of immune responses including T cell proliferation and activation, macrophage, B cell, dendritic cell and NK cell functions. The large number of Treg cells in endometriotic lesions helps retrograde endometrial tissue fragments to escape the host immune surveillance system by reducing the recruitment of the above immune cells, thereby allowing the initiation and establishment of endometriosis (65). In summary, these compelling evidence from the literatures support the opinion that immune cells recruited by the newly generated lymphatic vessels are advancing the development of endometriosis.

Other than the above-mentioned functions that can be performed by recruited immune cells, pro-inflammatory cytokines secreted by immune cells, can further boost the progression of endometriosis. The elevated concentrations of crucial pro-inflammatory cytokines, namely IL-1β, IL-6 and TNF-α, have been demonstrated in patients with endometriosis (66). Subsequent activation of the inflammatory response promotes the secretion of cytokines and chemokines in the peritoneal cavity, which forms a microenvironment that facilitates the development of ectopic endometrial tissue by contributing to local angiogenesis and disrupting the normal apoptotic process (67). The IL‐1 family cytokines are the secretory macrophage products, with IL‐1β being the most important mediator of acute and chronic inflammation and immune response. Defective mechanisms involved in the control of local IL‐1β activity may enhance susceptibility for the adhesion and growth of the ectopic endometrium and it leads to the development of endometriosis (68). IL-6 cytokine is considered to be the promoter of many biological activities. The ability of IL-6 to manage the survival, proliferation, and also the differentiation of cells is the reason why this interleukin may act as pro- and anti-inflammatory mediator (69). Higher levels of expressed IL-6 were measured in the peritoneal fluid and serum from women with endometriosis (70). In a similar study of reference, the expression rate of IL-6 was also higher in patients with endometriosis and it had a dependence on the stage of the disease (higher in III/IV vs. I/II stage) (71). TNF-α is a secretory factor of active macrophages known to have potent inflammatory cytotoxic and angiogenic characteristics. Several studies have demonstrated higher concentration of TNF-α in the peritoneal fluid of women with endometriosis than those without, as well as a direct correlation between TNF-α level and disease severity. Besides, elevation of TNF-α in peritoneal fluids is associated with infertility induced by endometriosis (72, 73). In brief, based on the function of immune cells and corresponding factors recruited by new lymphatic vessels, it is reasonable to conclude that BST2-mediated immune regulation can contribute to the development of endometriosis.

Noticeably, the well-known proinflammatory cytokines IL-1β and TNF-α play positive roles in activating the NF‐κB pathway and the expression of downstream target genes, respectively. This means that a vicious cycle is involved in the development of endometriosis. Newly generated lymphatic vessels elicit higher production of the proinflammatory factor IL-1β, which mediates greater lymphangiogenesis by activating the NF-κB pathway, which in turn leads to more newly generated lymphatic vessels (50, 58, 74). In other words, the regulation of NF‐κB involves positive feedback through the NF‐κB–mediated synthesis of IL-1β and TNF-α (75). Our study unveiled the mechanism of lymphangiogenesis in endometriotic lesions and further provided a potential diagnostic or therapeutic strategy for endometriosis.

**Figure 13:**
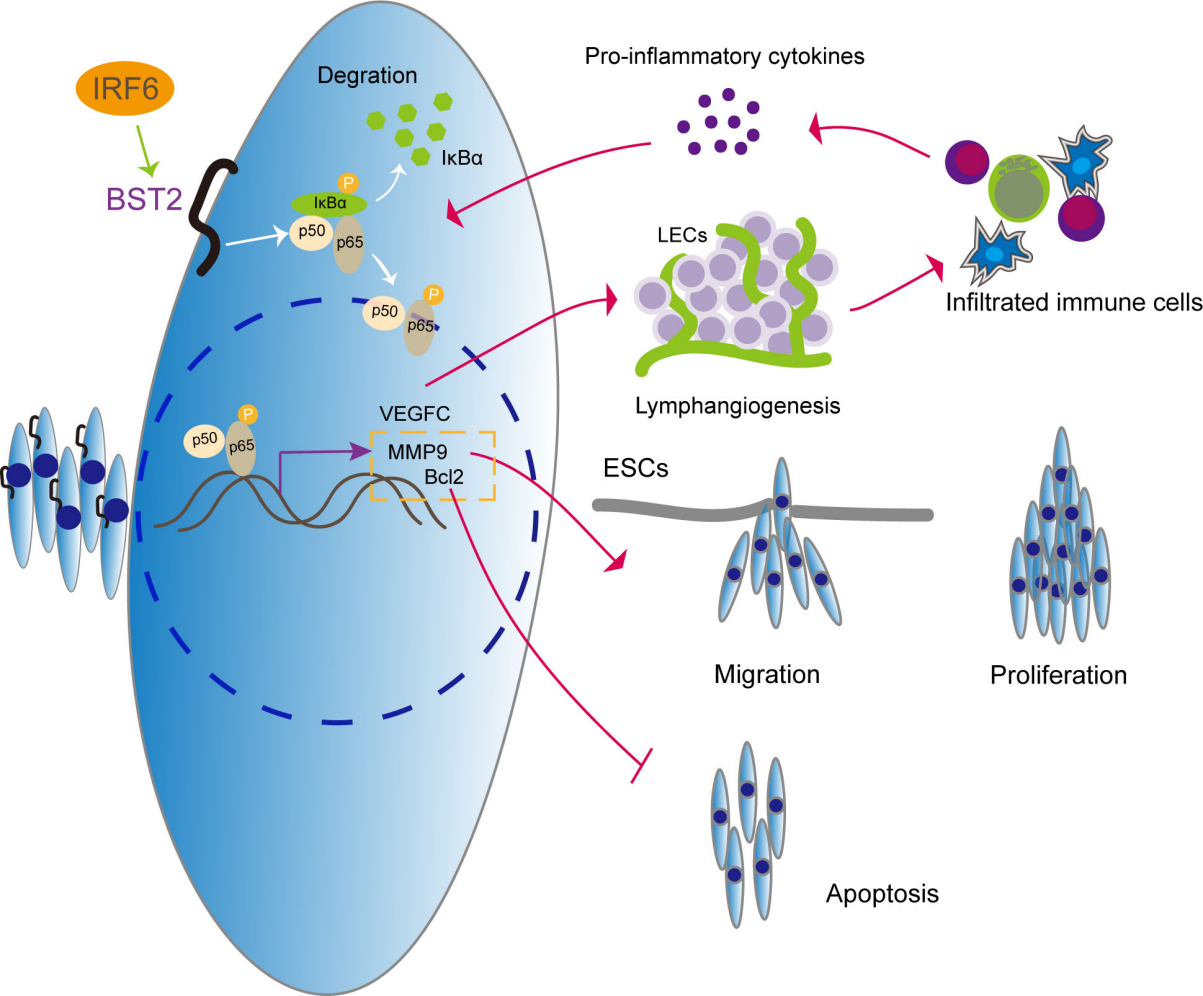
The illustrative model of BST2 in the development of endometriosis.

Overall, the data presented in this study reveal the transcription factor IRF6-associated regulation of BST2 expression, the pathological function of BST2 in endometriosis, novel signaling pathway mediated by BST2, and the therapeutic potential of targeting BST2 for the treatment of endometriosis (**Figure 13**). To our knowledge, this is the first report to thoroughly characterize the regulation, signaling, function, and therapeutic potential of BST2 in endometriosis. It is anticipated that targeting BST2 could be an alternative, non-hormonal treatment for endometriosis in the future.

## 5 Conclusion

IRF6 regulates the high expression of BST2, which further activates the NF-κB signaling pathway to induce proliferation, migration, apoptosis and lymphangiogenesis in endometriosis. More importantly, we explore the vicious cycle that is formed by infiltrating immune cells in new lymphatic vessels, which elicit the production of the proinflammatory cytokine IL-1β, which acts as an NF-κB activator, and promotes lymphangiogenesis *via* NF-κB activation.

## Data availability statement

The original contributions presented in the study are included in the article/**Supplementary Material**. Further inquiries can be directed to the corresponding author.

## Ethics statement

The studies involving human participants were reviewed and approved by The Ethics Committee of First Affiliated Hospital of Harbin Medical University. The patients/participants provided their written informed consent to participate in this study. The animal study was reviewed and approved by The Animal Experimental Ethics Committee of the First Affiliated Hospital of Harbin Medical University.

## Author contributions

GZ designed the research. JL and YH conducted the data processing and experimental analysis. YQ and CR collected the clinical samples. YC, XW, LS and XZ processed data. All authors contributed to the article and approved the submitted version.

## Funding

This study was supported by the National Natural Science Foundation of China (grant number 81971359); Natural Science Foundation of Heilongjiang Province (grant number LH2019H027); the Heilongjiang Postdoctoral Program Foundation (LBH-Z19085) and the Outstanding Young Medical Talents Training Fund project of the First Affiliated Hospital of Harbin Medical University (HYD2020YQ0021).
