# Correction: Promotion of BST2 expression by the transcription factor IRF6 affects the progression of endometriosis

**DOI:** 10.3389/fimmu.2025.1748838

**Published:** 2025-12-23

**Authors:** Jixin Li, Yanan He, Yanjun Qu, Chengcheng Ren, Xiaotong Wang, Yan Cheng, Liyuan Sun, Xin Zhang, Guangmei Zhang

**Affiliations:** 1 Department of Gynecology, The First Affiliated Hospital of Harbin Medical University, Harbin, Heilongjiang, China; 2 Central Laboratory, The First Affiliated Hospital of Harbin Medical University, Harbin, Heilongjiang, China

**Keywords:** endometriosis, transcription factor, immune-related genes, NF‐κB, lymphangiogenesis, feedback loop

There was a mistake in **Figure 3B** as published. Two small images (a and b) were placed in the wrong position.

The corrected **Figure 3B** appears below. The positions of the two images have been swapped and updated.

Also, due to figure compression, the quality of the Western blot images for β-tubulin in **Figure 2F** and actin in **Figure 6A** was compromised. To avoid misleading readers, we have uploaded high-quality versions of these two WB images as requested.

**Figure 2:**
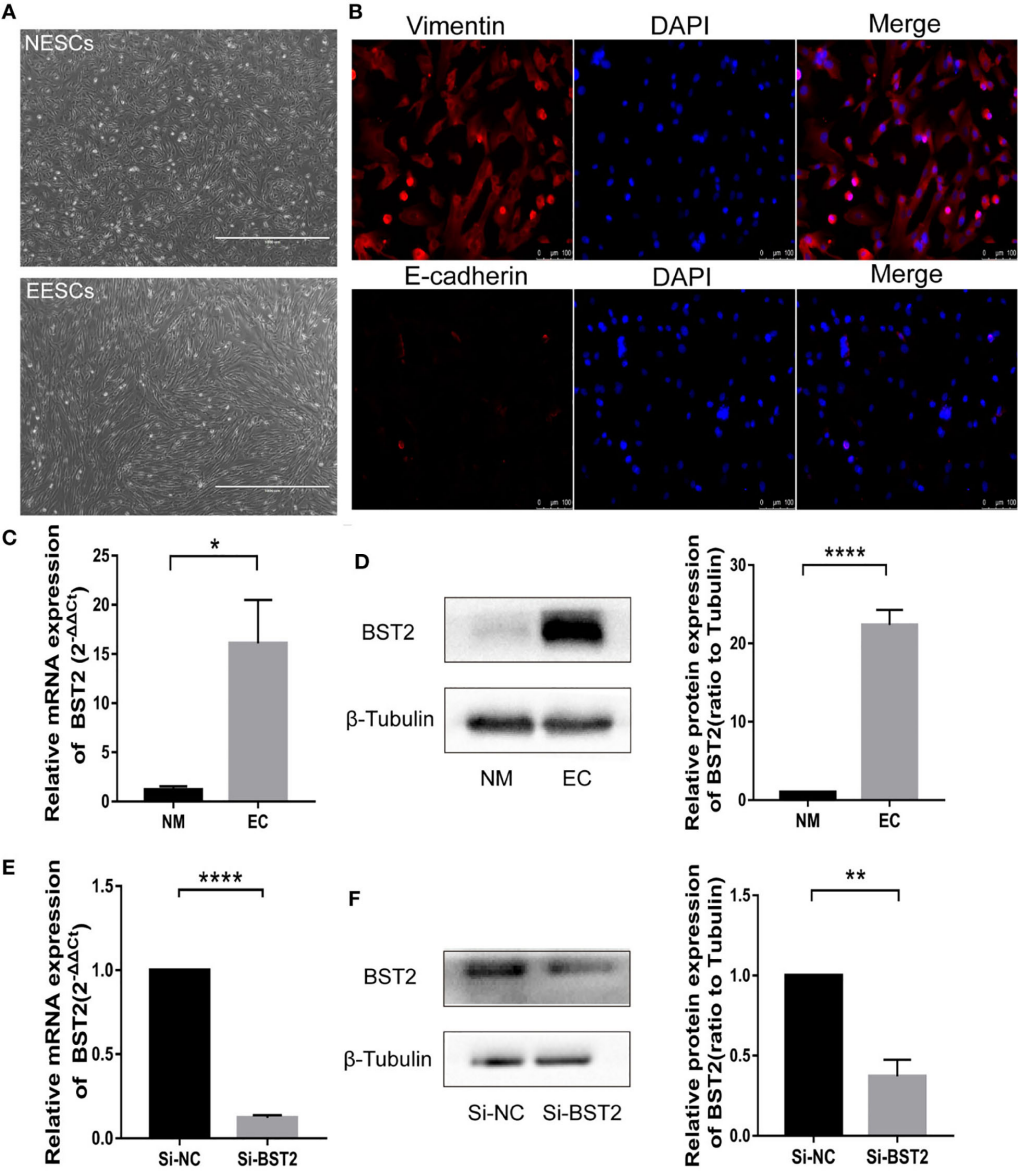
Identification of endometrial stromal cells and BST2 expression at the cellular level. **(A, B)**, The identification of endometrial stromal cells on the morphology **(A)** and Immunofluorescence **(B)**. **(C, D)**, BST2 mRNA **(C)** and protein **(D)** expression at the cellular level. **(E, F)**, Identification of the transfection efficiency of Si-BST2 at the mRNA **(E)** and protein **(F)** levels. *p < 0.05; **p < 0.01; ****p < 0.0001.

**Figure 3:**
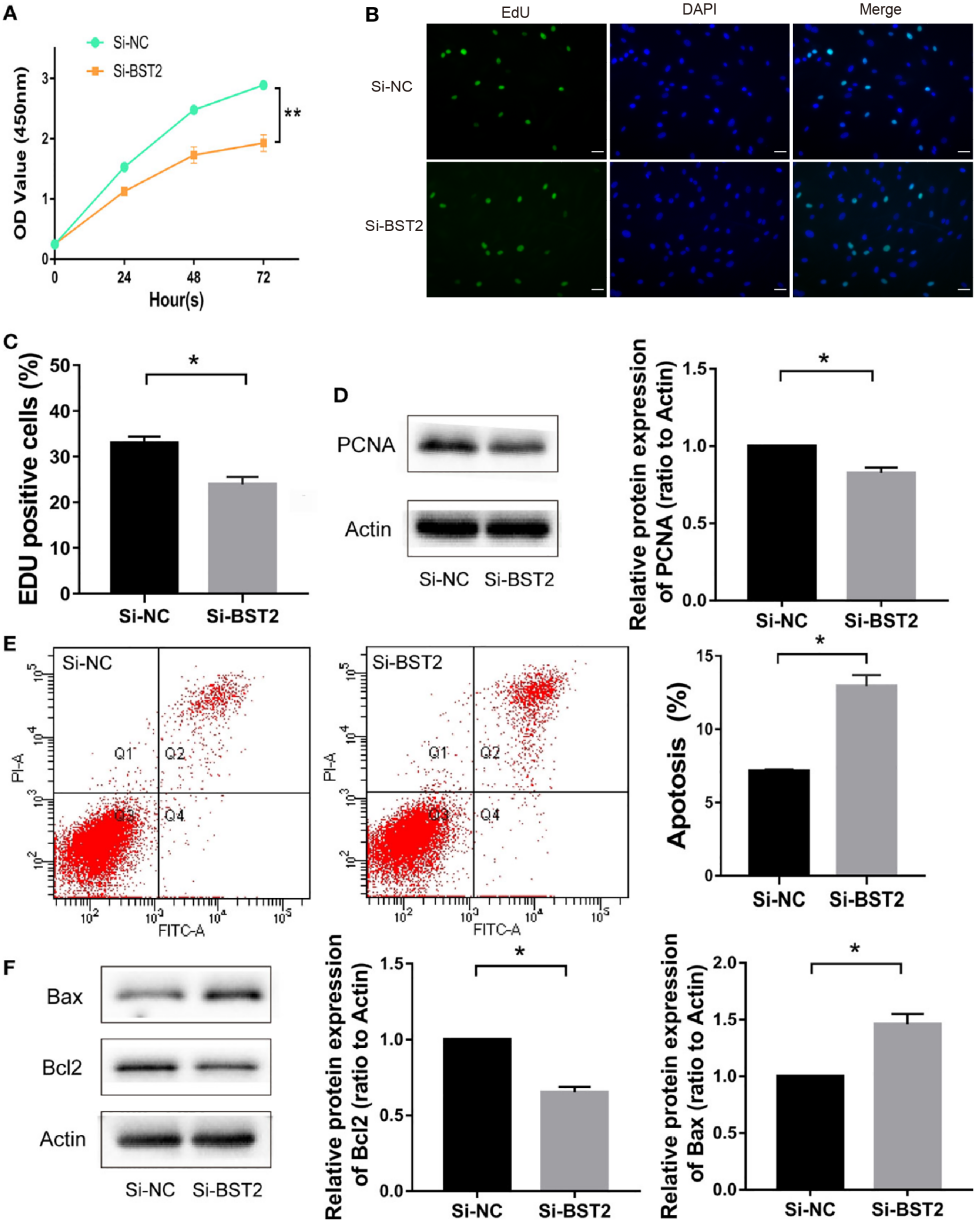
The effect of BST2 on cell proliferation and apoptosis. **(A–C)**, The proliferation of EESCs was examined by CCK-8 assay **(A)** and EdU **(B, C)** after Si-BST2 or Si-NC treatment for 48 h. **(D)**, The proliferation-related protein PCNA was measured by Western blot assay after Si-BST2 or Si-NC treatment for 48 h. **(E)**, The Flow cytometry was performed to detect the apoptosis rates of the EESCs transfected with Si-BST2 or Si-NC. **(F)**, Western blot analysis was used to measure the levels of Bax and Bcl2 in cells transfected with Si-BST2 or Si-NC. *p < 0.05; **p < 0.01.

**Figure 6:**
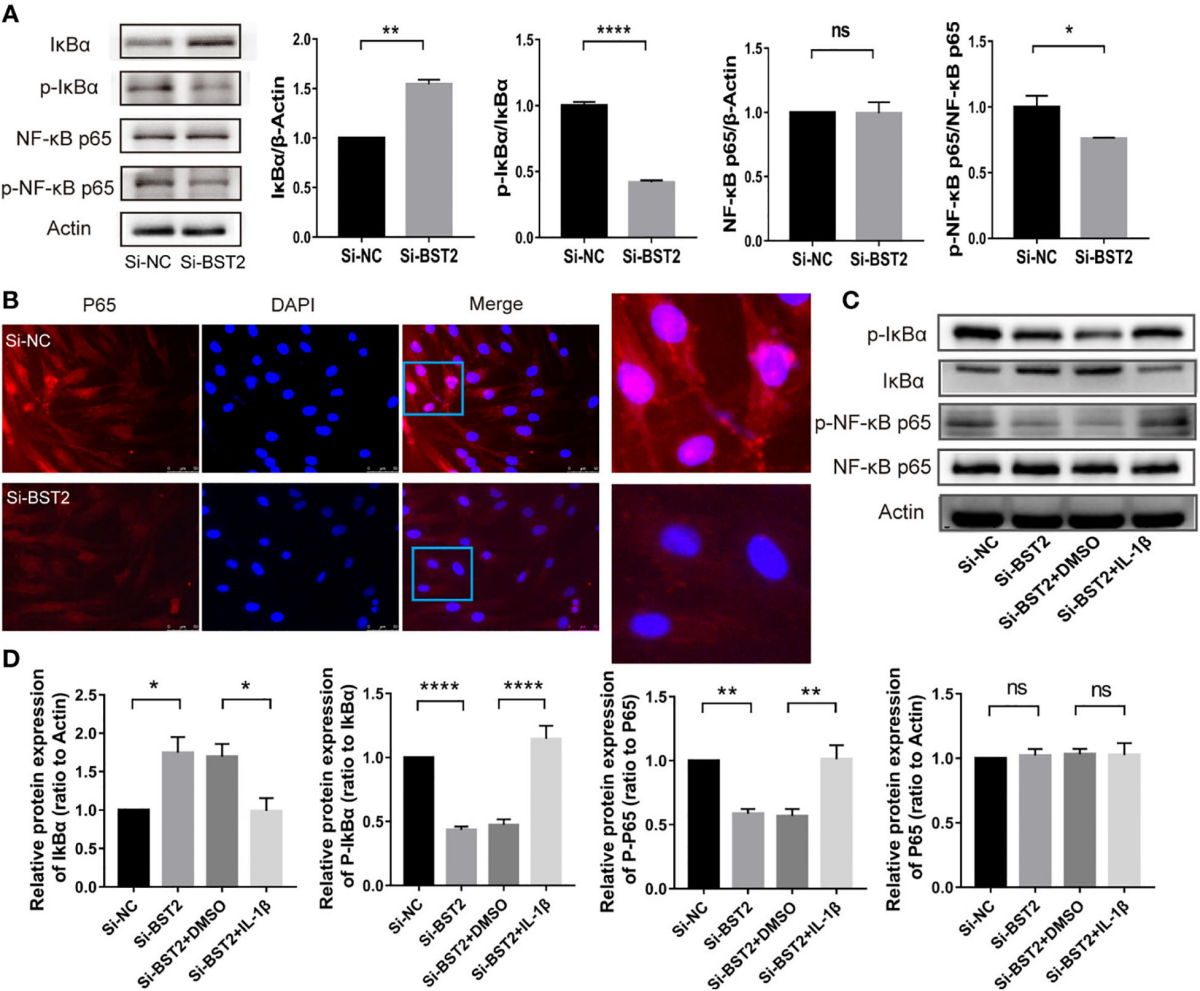
BST2 activated the NF-κB signaling pathway. **(A)**, The expressions of key proteins of NF-κB pathway were measured by western blot analysis after transfection with Si-BST2 or Si-NC for 48h. **(B)**, The Representative images of Immunofluorescence showed the cellular localization of NF-κB p65 protein in EESCs after transfection with Si-BST2 or Si-NC for 48h. **(C, D)**, The protein expressions of NF-κB pathway were measured by western blot analysis after treatment with Si-BST2 or Si-NC or/and the NF-κB pathway activator IL-1β. ns, p ≥ 0.05; *p < 0.05; **p < 0.01; ****p < 0.0001.

High-quality raw image of β-tubulin in **Figure 2F**:

High-quality raw image of actin in **Figure 6A**:

The original version of this article has been updated.

