# Supplementary material for: Promotion of BST2 expression by the transcription factor IRF6 affects the progression of endometriosis
Source: Front Immunol. 2023 Apr 18;14:1115504. doi: 10.3389/fimmu.2023.1115504 (PMC10151653; doi:10.3389/fimmu.2023.1115504)
Supplement: Supplementary file 1 [file Table_1.docx]

Table S1 Primer sequences for qRT-PCR

| **Gene** | **Primer sequences** |
| --- | --- |
| BST2 | Forward: ACACTGTGATGGCCCTAATG  Reverse: CGTCCTGAAGCTTATGGTTTAATG |
| IRF6 | Forward: GCCACCGTTTGAGATCTACTT  Reverse: CCACTACTGGAATGACCTGAAC |
| GAPDH | Forward: CTGGGCTACACTGAGCACC  Reverse: AAGTGGTCGTTGAGGGCAATG |

Table S2 Primary antibodies for Western blot assay

| **Antibody name** | **Dilutions** | **Vendor name** | **Cat No.** |
| --- | --- | --- | --- |
| BST2 | 1:1000 | Abcam | ab243230 |
| IRF6 | 1:1000 | ABclonal | A3209 |
| PCNA | 1:1000 | Abcam | ab29 |
| MMP2 | 1:1000 | Abcam | 92536 |
| MMP9 | 1:1000 | Abcam | 76003 |
| Bcl2 | 1:1000 | Abcam | 32124 |
| Bax | 1:1000 | Abcam | 32503 |
| VEGFC | 1:1000 | Proteintech | 67116-1-Ig |
| IκBα | 1:1000 | Abcam | 32518 |
| P- IκBα | 1:1000 | Abcam | 92700 |
| P65 | 1:1000 | Abcam | 32536 |
| P-P65 | 1:1000 | Abcam | 76302 |
| Tubulin | 1:5000 | Proteintech | 66240-1-Ig |
| β-actin | 1:5000 | Proteintech | 66009-1-Ig |
| GAPDH | 1:5000 | Proteintech | 60004-1-Ig |
